# Crystallographic Aspects, Photophysical Properties, and Theoretical Survey of Tetrachlorometallates of Group 12 Metals [Zn(II), Cd(II), and Hg(II)] with a Triply Protonated 2,4,6-Tris(2-pyridyl)-1,3,5-triazine Ligand

**DOI:** 10.1021/acs.inorgchem.2c04521

**Published:** 2023-05-02

**Authors:** Samit Pramanik, Sumanta Jana, Kinsuk Das, Sudipta Pathak, Joaquin Ortega-Castro, Antonio Frontera, Subrata Mukhopadhyay

**Affiliations:** † Department of Chemistry, Jadavpur University, Kolkata 700032, India; ‡ Department of Chemistry, Chandernagore College, Hooghly, West Bengal 712136, India; § Department of Chemistry, Haldia Government College, Debhog, Purba Medinipur, West Bengal 721657, India; ∥ Department of Chemistry, Universitat de les IllesBalears, Crta. de Valldemossa km 7.5, 07122 Palma de Mallorca (Baleares), Spain

## Abstract

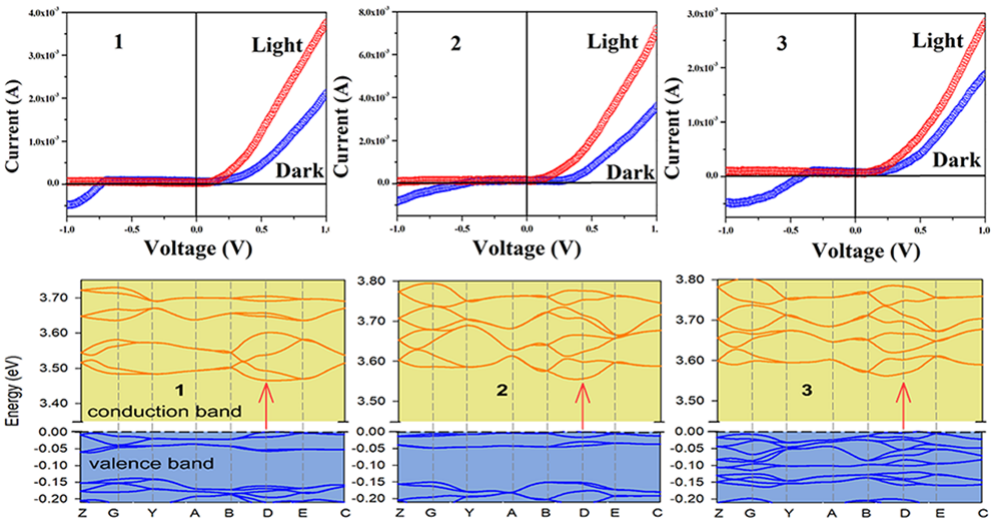

Zn(II) (complex **1**), Cd(II) (complex **2**), and Hg(II) (complex **3**) complexes have been synthesized using a triply protonated tptz (H_3_tptz^3+^) ligand and characterized mainly by single-crystal X-ray analysis. The general formula of all of the complexes is (H_3_tptz)^3+^·Cl^–^·[MCl_4_]^2–^·*n*H_2_O (where *n* = 1, 1.5, and 1.5 for complexes **1**, **2**, and **3**, respectively). The crystallographic analysis reveals that the anion···π, anion···π^+^, and several hydrogen bonding interactions play a fundamental role in the stabilization of the self-assembled architectures that in turn help to enhance the dimensionality of all of the complexes. In addition, Hirshfeld surfaces and fingerprint plots have been deployed here to visualize the similarities and differences in hydrogen bonding interactions in **1–3**, which are very important in forming supramolecular architectures. A density functional theory (DFT) study has been used to analyze and rationalize the supramolecular interactions by using molecular electrostatic potential (MEP) surfaces and combined QTAIM/NCI plots. Then, the device parameters for the complexes (**1**–**3**) have been thoroughly investigated by fabricating a Schottky barrier diode (SBD) on an indium tin oxide (ITO) substrate. It has been observed that the device made from complex **2** is superior to those from complexes **1** and **3**, which has been explained in terms of band gaps, differences in the electronegativities of the central metal atoms, and the better supramolecular interactions involved. Finally, theoretical calculations have also been performed to analyze the experimental differences in band gaps as well as electrical conductivities observed for all of the complexes. Henceforth, the present work combined supramolecular, photophysical, and theoretical studies regarding group 12 metals in a single frame.

## Abstract 2

We report here the syntheses, X-ray crystal structures, and supramolecular aspects of tetrachlorometallates covering group 12 metals derived from a triply protonated 2,4,6-tris(2-pyridyl)-1,3,5-triazine ligand along with their photophysical properties in a single frame.

## Introduction

Recent years have witnessed an immense growth in crystal engineering propelled by numerous noncovalent interactions.^1,2^ The primary objective of the crystal engineering is to achieve the preferred structural topology that requires judicious choice of ligands and metal precursors.^3,4^ Based on crystallographic data available in the structural database, new supramolecular synthons have been planned and synthesized accordingly. In general, either cationic or anionic part of the complex interacts with oppositely charged counterions by exploiting several noncovalent interactions including π···π, cation···π, anion···π, hydrogen bonding interactions, etc.^5−7^ Second-sphere coordination binding occurs using noncovalent interactions of chemical species to the first-sphere coordination (covalently bonded) of a transition metal complex. In current research, supramolecular interactions directed by organic cations have arrested interest due to their unique structural features related to stacking interactions.^8,9^ The second-sphere coordination strategy can form a binding site for anion recognition selectively and effectively.^10,11^ In this regard, some organic–inorganic hybrid complexes can be prepared by the second-sphere coordination strategies involving organic cations and anionic metal complexes. 2,4,6-Tris(2-pyridyl)-1,3,5-triazine (tptz) or its structural analogues have been well recognized as neutral binding motifs and can be used as primary as well as auxiliary ligands in contemporary metallo-supramolecular chemistry.^12−16^ Group 12 metal ions in the periodic table have a d^10^ electronic configuration and do not exhibit any crystal field stabilization, and hence, their geometrical preference can be guided by steric factors mainly. As both “π” and “anion” are electron-rich species, their interactions have been unacknowledged for a long period. In 2002, Alkorta et al., Deyà et al., and Mascal et al. confirmed theoretically the presence of favorable noncovalent interactions between electronic-deficient aromatic rings and anions.^17−19^ From the crystallographic database, it was found that only anion···π interaction was not able to ensure structural assembly, but an orchestrated interplay between either anion···π and π···π or anion···π and hydrogen bonding (classical, nonclassical, or both) interactions was energetically favorable to construct desired supramolecular architectures.^6−8^

Metal–organic hybrids with essentially high conducting properties have grown to be an important research topic owing to their potential applications in electronic devices. Designing such flexible metal–organic hybrids of organic and inorganic building blocks into stable structural integrity plays a significant role in electron transportation, which in turn displays a wide spectrum of applications as a Schottky barrier diode (SBD) in electronics and optoelectronic devices.^20,21^ An SBD is broadly used in photovoltaics,^22^ batteries,^23,24^ sensors,^25^ supercapacitors,^26^ and transistors.^27^ Despite these advantages, application of most of the metal–organic hybrids is not up to the mark because of incompetent electron transportation (lower conductivity lesser than 10^–10^ S/cm) between insulated organic ligands and non-redox metal ions.^28,29^ Thus, exploring the role of metals in metal–organic hybrids with enhanced conductivity has become a trendy research topic for investigators. However, there are other device parameters like a short lifetime, high barrier potential and transit time, low effective mobility and carrier concentration, etc., that must be confirmed before fabricating the final device. The flexibility of devices depends on the assortment of metal ions and the binding functionality of the ligands. Therefore, before preparing metal–organic hybrids, one has to understand the influence of weak interactions in metal–organic frameworks (MOFs) for easy electron tunneling. Even weak interactions can increase electrical conductivity by several orders (by electronic delocalization), and linkers with electron-donating or -withdrawing groups convey more interactions for making electronically conductive arrangements.^30^ By precisely controlling these supramolecular interactions, a chemist can impart new functions such as hydrophobicity, high thermal stability, charge carrier density, and charge mobility related to these materials. Li et al. and Dutta et al. successfully fabricated SBD devices with d^10^ metal ions (Zn^2+^, Cd^2+^) by using carboxylate linkers.^31,32^

Considering the above facts, we report here the syntheses, X-ray crystal structures, and supramolecular aspects of Zn (**1**), Cd (**2**), and Hg (**3**) complexes derived from a triply protonated 2,4,6-tris(2-pyridyl)-1,3,5-triazine ligand along with their photophysical properties.

In this present work, we have strategically planned to restrict the ligand “tptz” not to participate as a chelating ligand to form a metal complex but should act as an organic cation. In this regard, we have protonated all of the peripheral pyridine nitrogen (to restrict the neutral coordination in the first coordination sphere), maintaining the proper acidic pH of the reaction medium. Taking into consideration our previous works,^6,9^ where the title ligand was monoprotonated (pH was adjusted to 4), herein, we regulated the pH to 0 (i.e., 1 M acidity) for further protonation of the ligand as our requirement. To stabilize this almost planar large-size cation (with more than one positive charge), our intention was to generate a large-size anion preferably a tetrahedral-like tetrahalometallate having more than one negative charge.

In this regard, we have chosen group 12 metal ions (Zn^2+^, Cd^2+^, and Hg^2+^) that are able to form tetrachlorometallates [MCl_4_]^2–^ (M = Zn, Cd, Hg) in the presence of dilute HCl (Scheme S1). Herein, the supramolecular interactions between the organic salt (tptzH_3_)^3+^ and the tetrachlorometallate having the general formula (H_3_tptz)^3+^·Cl^–^·[MCl_4_]^2–^·*n*H_2_O are described. The geometry of the central metal in the complexes is distorted to be tetrahedral. Here, the tptz ligand is of our prime interest due to its large π-conjugation, and we want to utilize these π-based supramolecular interactions to execute extended architectures. Hence, in this communication, we have found hydrogen bonding and anion···π interactions in all these complexes. The interesting water–anion clusters have been explored for the title complexes (**1–3**) in this work. Structural analysis supported by Hirshfeld surfaces and fingerprint plots has also been displayed to gain additional insights into the hydrogen bonding interactions for all these complexes. The structure-directing role of anion−π/π^+^ and hydrogen bonding interactions has been analyzed by DFT calculations. Under illumination conditions, the magnitudes of the photophysical properties of the complexes improve remarkably, though the improvement differs from complex to complex, which has also been analyzed theoretically. To our knowledge, it is the first report covering three group 12 metals that encompass both supramolecular and photophysical studies with proper theoretical justification.

## Experimental Section

The details of starting materials and physical measurements have been incorporated in the Supporting Information.

### Synthesis of [H_3_tptz]Cl·[ZnCl_4_]·H_2_O (Complex **1**)

An aqueous solution (5 mL) of ZnCl_2_ (0.136 g, 1 mmol) was added dropwise to a stirred warm solution (40 °C) of 2,4,6-tris(2-pyridyl)-1,3,5-triazine (0.312 g, 1 mmol) dissolved in 10 mL of HCl (1 M). After constant stirring for 2 h, the resulting solution was filtered and the filtrate was left for slow evaporation without any disturbance. Crystals of **1** suitable for X-ray data collection were obtained after two weeks (yield: 73%). Anal. calcd for C_18_H_15_Cl_5_N_6_Zn·H_2_O: C, 37.53; H, 2.97; N, 14.59. Found: C, 37.50; H, 2.94; N, 14.56%. Main Fourier transform infrared (FT-IR) absorptions, (KBr, cm^–1^): 3458(vs), 3376(vs), 3055(s), 2606(w), 2068(w), 2008(w), 1652(vs), 1615(vs), 1543(vs), 1525(s), 1396(vs),1366(s), 1342(s), 1313(w) (Figure S1).

### Synthesis of [H_3_tptz]Cl·[CdCl_4_]·1.5H_2_O (Complex **2**)

Complex **2** was synthesized using a similar procedure as that of complex **1**. Here, an aqueous solution of CdCl_2_·H_2_O (0.201 g, 1 mmol) was used instead of ZnCl_2_. After three weeks, pale yellow block-shaped single crystals of **2** were found by slow evaporation of the solvent (yield: 72%). Anal. calcd for C_18_H_15_Cl_5_N_6_Cd.1.5H_2_O: C, 33.72; H, 2.99; N, 13.11. Found: C, 33.69; H, 2.94; N, 13.08%. Main FT-IR absorptions, (KBr, cm^–1^): 3558(s), 3498(vs), 3412(vs), 3081(s), 3055(s), 2517(w), 2069(s), 2000(s), 1940(w), 1643(vs), 1616(vs), 1605(s), 1546(s), 1538(s), 1521(s), 1440(s), 1396(vs), 1366(s), 1344(s), 1300(s) (Figure S1).

### Synthesis of [H_3_tptz]Cl·[HgCl_4_]·1.5H_2_O (Complex **3**)

Complex **3** was obtained via the same procedure as that for complex **1** using an aqueous solution of HgCl_2_ (0.271 g, 1 mmol) instead of ZnCl_2_. The yellow crystals of **3** suitable for X-ray analysis were collected after one month with a yield of 67%. Anal. calcd for C_18_H_15_Cl_5_N_6_Hg.1.5H_2_O: C, 30.02; H, 2.52; N, 11.67. Found: C, 29.99; H, 2.48; N, 11.65%. Main FT-IR absorptions, (KBr, cm^–1^): 3562(S), 3506(s), 3380(vs), 3242(s), 3055(s), 2518(w), 2067(s), 2005(s), 1643(s), 1615(vs), 1545(s), 1523(s), 1439(s), 1396(vs), 1366(s), 1344(s) (Figure S1).

Details of X-ray crystallographic analysis, Hirshfeld surface analysis, and theoretical methods have been provided in the Supporting Information.

## Results and Discussion

### Structural Description with Comparison for Complexes **1**, **2**, and **3**

The molecular structures with an atom numbering scheme for all of the complexes are shown in Figure S2. All these complexes are isostructural and bear monoclinic structures with the *P*2_1_/*n* space group. All of the complexes contain a tetrachlorometallate MCl_4_^2–^ (where M = Zn, Cd, and Hg) unit, a noncoordinated chloride ion, and a triply protonated organic moiety (H_3_tptz^3+^) to balance the entire charge. The product stoichiometry is thus given by the overall charge balance with H_3_tptz^3+^/MCl_4_^2–^/Cl^–^= 1:1:1. The phase purity of all of the complexes has been confirmed by the powder X-ray diffraction (PXRD) pattern analysis (Figure S3). All three complexes have been hydrated to different extents. Complex **1** bears one H_2_O molecule, whereas complexes **2** and **3** bear one and a half (1.5) H_2_O molecules per asymmetric unit. Thermogravimetric (TG) analysis was also carried out for all three complexes (**1–3**) to confirm the number of water molecules present in the complexes (Figure S4). Using the water molecules, complex **1** forms a one-dimensional (1D) water–anion cluster, whereas complexes **2** and **3** form similar types of both 1D and two-dimensional (2D) water–anion clusters.

**Figure 1:**
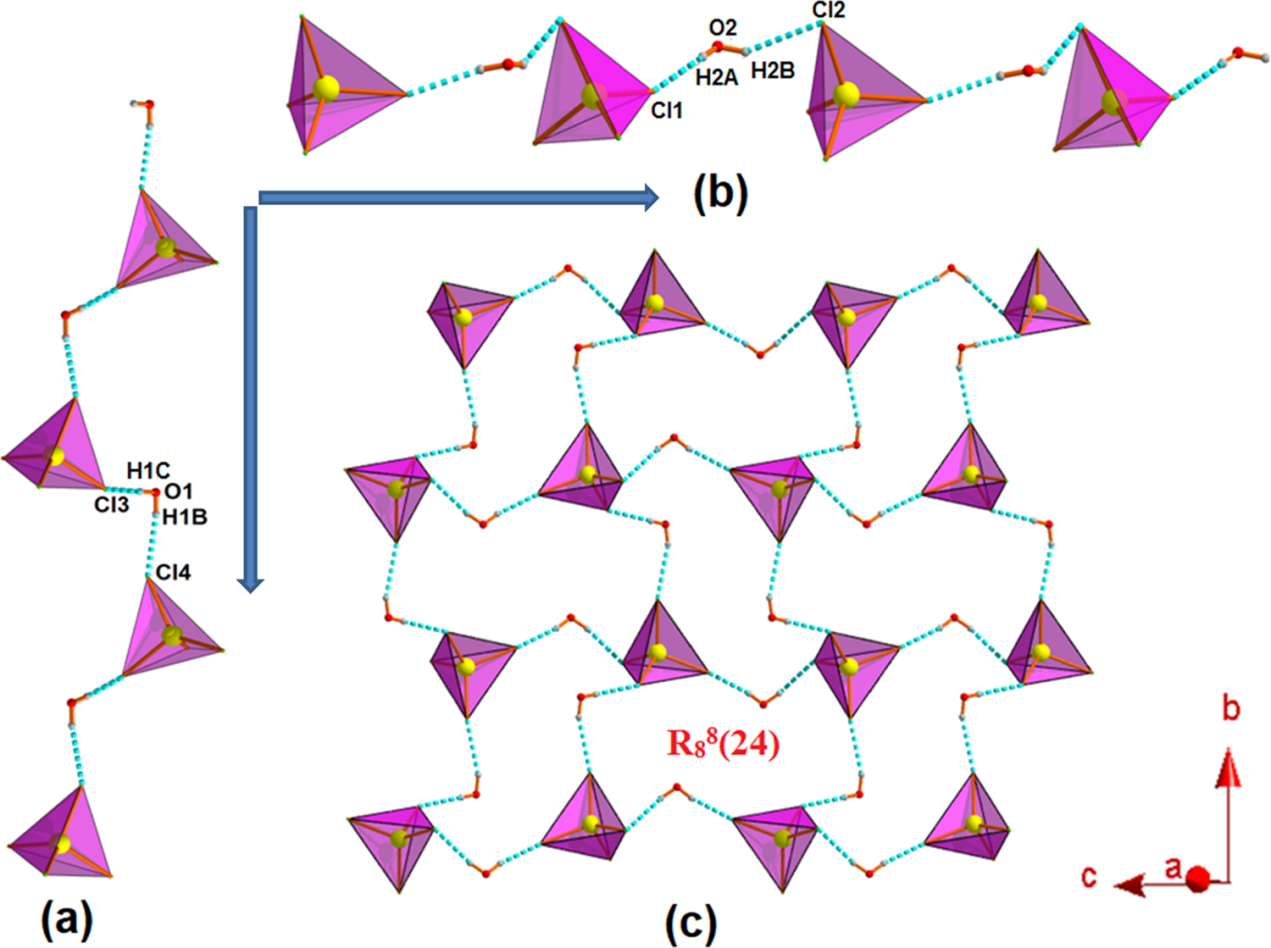
(a) Formation of a 1D water–anion cluster along the (010) direction. (b) Propagation of another 1D water–anion cluster along the (001) direction. (c) Extension of water–anion cluster in the *bc*-plane in complex **2**.

In complex **1**, the noncoordinated water molecule acts as a double donor to Cl(1) and Cl(4) of two different ZnCl_4_^2–^ ions at angles of 166 and 179°, respectively, to produce a 1D zigzag chain along the (010) direction (Figure S5). In complex **2**, two different noncoordinated water molecules are engaged with CdCl_4_^2–^ ions in producing two different 1D water–anion clusters (Figure 1a,b) that in turn make an esthetically beautiful 2D extended architecture having an R_8_^8^(24) ring motif in the “*bc*”-plane (Figure 1c).

A similar type of water–anion cluster (Figure 1) has also been observed for complex **3**, where HgCl_4_^2–^ ions are hydrogen-bonded with two different noncoordinated water molecules present in it (Figure S6).

In our previous studies,^6,9^ we were able to synthesize Ni(II) complexes with the same title ligand in its monoprotonated form (Htptz^+^), and in all these complexes, the metal ion was directly coordinated to Htptz^+^ and several supramolecular interactions are responsible to stabilize the structures. But numerous attempts to prepare single crystals (X-ray quality) with group 12 elements with the same protonated ligand in different solvents proved unsuccessful. We then strategically modified the synthetic protocol using further the protonated (here, triply) tptz ligand. Tetrahedral MCl_4_^2–^ ions (where M = Zn, Cd, and Hg) have got some stabilities from a stereo-electronic point of view (as all M^2+^ is the d^10^ system and devoid of any crystal field stabilization energies). With this contention, we paved our work in further protonation of the tptz ligand in relatively higher acidic conditions (here, we used 1 M acidity) to enhance the cationic charge and forcefully convinced it to interact with tetrachlorometallate ions. In this regard, our aim was to identify the role of supramolecular interactions in stabilization of the crystal structure in the solid state. The asymmetric units of complexes consist of M^2+^cations (M = Zn, Cd and Hg) coordinated to four chlorido ligands in tetrahedral fashion with ∠ClMCl ranging from 103.44(2) to 118.90(3)° (for complex **1**), 104.40(3) to 114.23(3)° (for complex **2**), and 103.72(8) to 114.98(9)° (for complex **3**). The M–Cl bond distances range from 2.2440(7) to 2.2897(7) Å (for complex **1**), 2.4397(7) to 2.4790(7) Å (for complex **2**), and 2.451(2) to 2.540(2) Å (for complex **3**). All M–Cl distances and ∠ClMCl angles are in good agreement with those reported earlier.^33−35^ The departure from the ideal tetrahedral angle (109°28′) is probably due to stereo-electronic relaxation as a demand of efficient crystal packing by improvising several supramolecular interactions.

Several kinds of hydrogen bonding interactions (both classical and nonclassical), namely, O–H···Cl, N–H···Cl, N–H···N, N–H···O, C–H···Cl, and C–H···O interactions, play a very crucial role in stabilizing the structures (Table S4). The local tetrahedral M^2+^ tetrachloro coordination sphere and noncoordinated chloride ions have participated in hydrogen bonding interactions and helped to generate higher dimensionality of the crystal structure in the solid state.

In the triply protonated organic moiety (Figure S7), the major coordination site is restricted due to two different intramolecular hydrogen bonding interactions (N1–H1···N2 and N6–H6···N2). The moderate coordination site is restricted through another intramolecular hydrogen bonding interaction (N5–H5···N4), but there are no such interactions in the minor coordination site.

**Figure 2:**
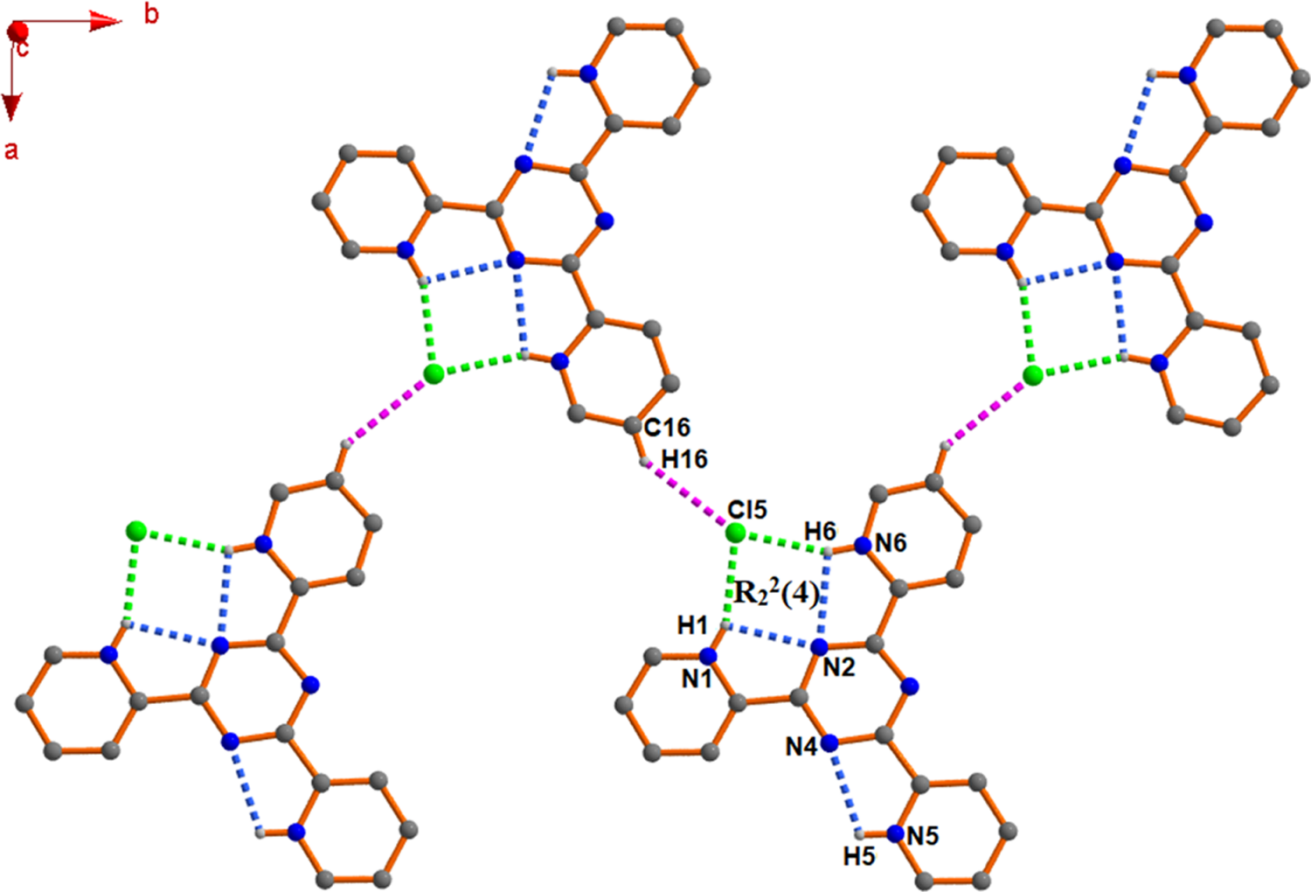
Propagation of a 1D zigzag chain along the (010) direction in complex **1**.

However, both protonated pyridine ring protons (N1–H1 and N6–H6) of the organic moiety further interact with the noncoordinated ionic chloride (Cl5) and generates an R_2_^2^(4) ring motif, which is propagated in a zigzag fashion along the (010) direction through the C16–H16···Cl5 interaction to produce a 1D chain (Figure 2).

The interesting phenomenon is that complexes **2** and **3** also exhibit the exact same architecture (Figure 2), where the noncoordinated chloride ion (Cl5) forms trifurcated hydrogen bonding interactions (N1–H1···Cl5, N6–H6···Cl5, and C16–H16···Cl5) to connect the triply protonated moieties.

On comprehensive analysis, it was exhibited that the 1D chain (shown in Figure 2) further extended its dimensionality (2D) through two different anion···π^+^ interactions, where the noncoordinated chloride ion (Cl5) simultaneously interacted with Cg(1) and Cg(3) [where Cg(1) and Cg(3) are the centroids of N1C1C2C3C4C5 and N5C9C13C12C11C10 rings, respectively] of the triply protonated moiety (H_3_tptz^3+^), as depicted in Figure S8. The same also occurred for complexes **2** and **3**.

The water–anion clusters play an important role to stabilize various supramolecular architectures for all three complexes in the solid state. In complex **1**, triply protonated organic moieties are almost parallel to each other with an intervening water–anion cluster (Figure S5), which connects these organic moieties through strong N5–H5···O1 interactions (1.94 Å) to ensure a 1D tape-like architecture (Figure S9) in the “*ab*”-plane. Another interesting phenomenon is that complexes **2** and **3** also exhibit architectures (Figures S10 and S11) similar to that of complex **1** (Figure S9), where triply protonated organic moieties are stitched with the water–anion cluster (as depicted in Figures 1a and S6a for complexes **2** and **3**, respectively).

Besides strong charge-assisted N–H···O interaction, weak nonclassical C–H···Cl–M hydrogen bonding contacts (between the aromatic ring hydrogen atom and the chloride ligands of the mononuclear complexes) further extend the dimensionality of the structures. In complex **1**, the protonated moieties are interlinked through a water–anion cluster (Figure S5) and three different C–H···Cl interactions [C2–H2···Cl3 (168°), C13–H13···Cl3 (153°), and C17–H17···Cl2 (150°)] to produce a 2D layer (Figure S12).

In complex **2**, the protonated organic moieties are arranged in such a fashion that the water–anion cluster (Figure 1b) connects these moieties by incorporating two different hydrogen bonding interactions [C10–H10···Cl3 (166°) and C17–H17···Cl2 (161°)], resulting in a different type of 2D architecture in the “*ac*”-plane (Figure S13). A similar 2D architecture (Figure S14) is also produced by the water–anion cluster (Figure S6b) for complex **3**.

Besides several hydrogen bonding interactions, all four chloride ions attached with group 12 metal ions have engaged in anion···π and anion···π^+^ interactions. Anion···π interactions are mediated by the middle triazine ring, whereas protonated peripheral pyridine rings of H_3_tptz^3+^ are responsible for anion···π^+^ interactions. When the aromatic π systems adopt positive charges, the charges are often observed to take part in anion···π^+^ interactions to strengthen the binding ability than that of conventional anion···π interactions. But, in this case, the anion···π interaction is stronger than the anion···π^+^ interaction. It was evident that generally, the central triazine ring participates in the anion···π interaction if the triazine ring is attached to some electron-withdrawing groups. Here, in our case, the central triazine ring is attached to three protonated pyridine rings that in turn make the central triazine ring sufficiently electron-deficient and dictate to take part in the strong anion···π interaction.

**Figure 3:**
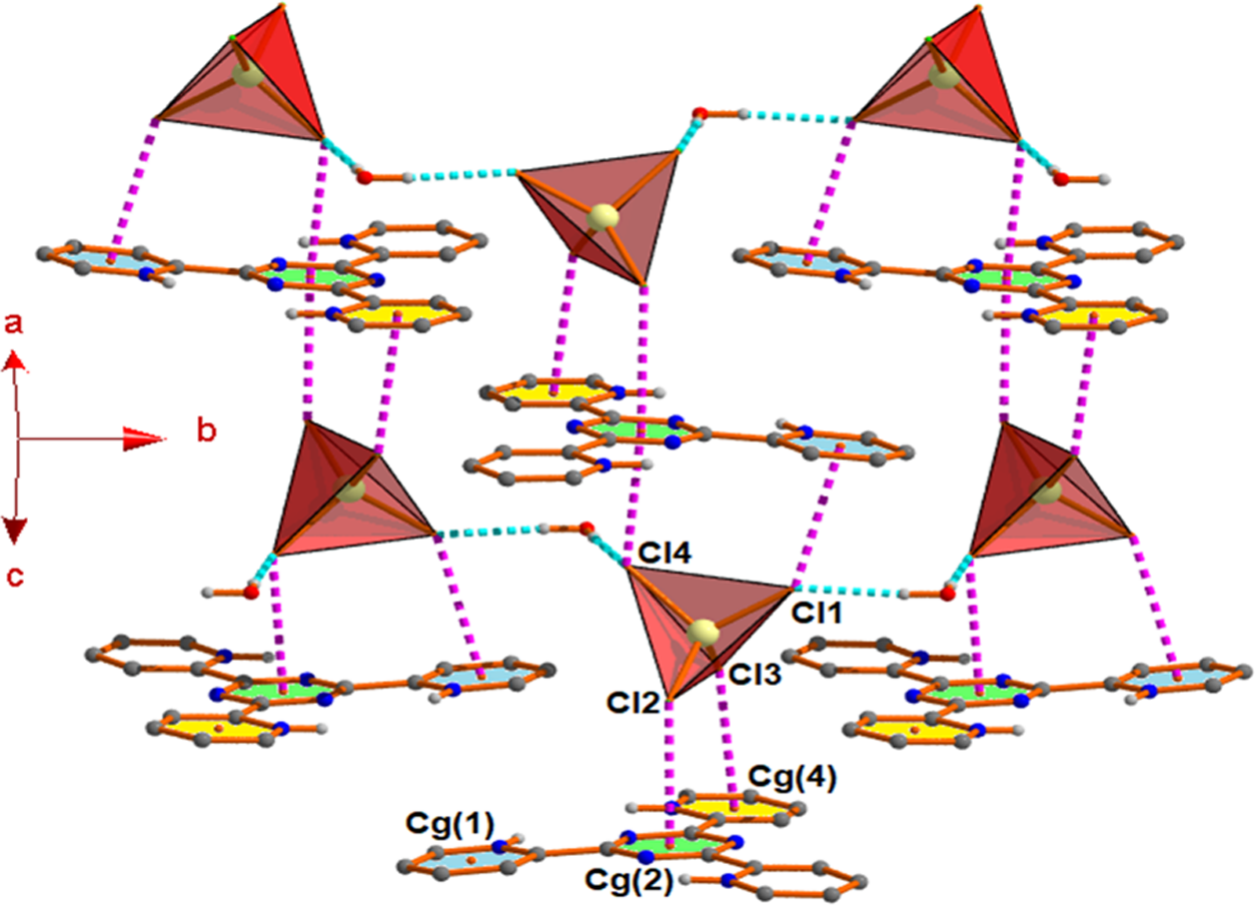
Perspective view of 2D architecture through water–anion cluster, anion···π/π^+^ interactions in complex **1**.

In complex **1**, Cl(2) and Cl(4) attached with Zn^2+^ form anion···π interactions with Cg(2), whereas Zn^2+^-bound Cl(1) and Cl(3) form anion···π^+^ interactions with Cg(1) and Cg(4) of the H_3_tptz^3+^ moiety [where Cg(1), Cg(2), and Cg(4) are the centroids of N1C1C2C3C4C5, N2C6N4C8N3C7, and N6C17C16C15C14C18 rings, respectively] to form a 1D chain (Figure 3). Finally, the noncoordinated water molecule connects ZnCl_4_^2–^ ions as a form of water–anion cluster (Figure S5) to extend the dimensionality to 2D in the *ab*-plane. The Zn–Cl···Cg bipodal anion···π/π^+^ interaction distances range from 3.3511(10) to 3.5829(12) Å and the corresponding angles are 123.16(3) and 135.84(3)°, respectively (Table S5).

**Figure 4:**
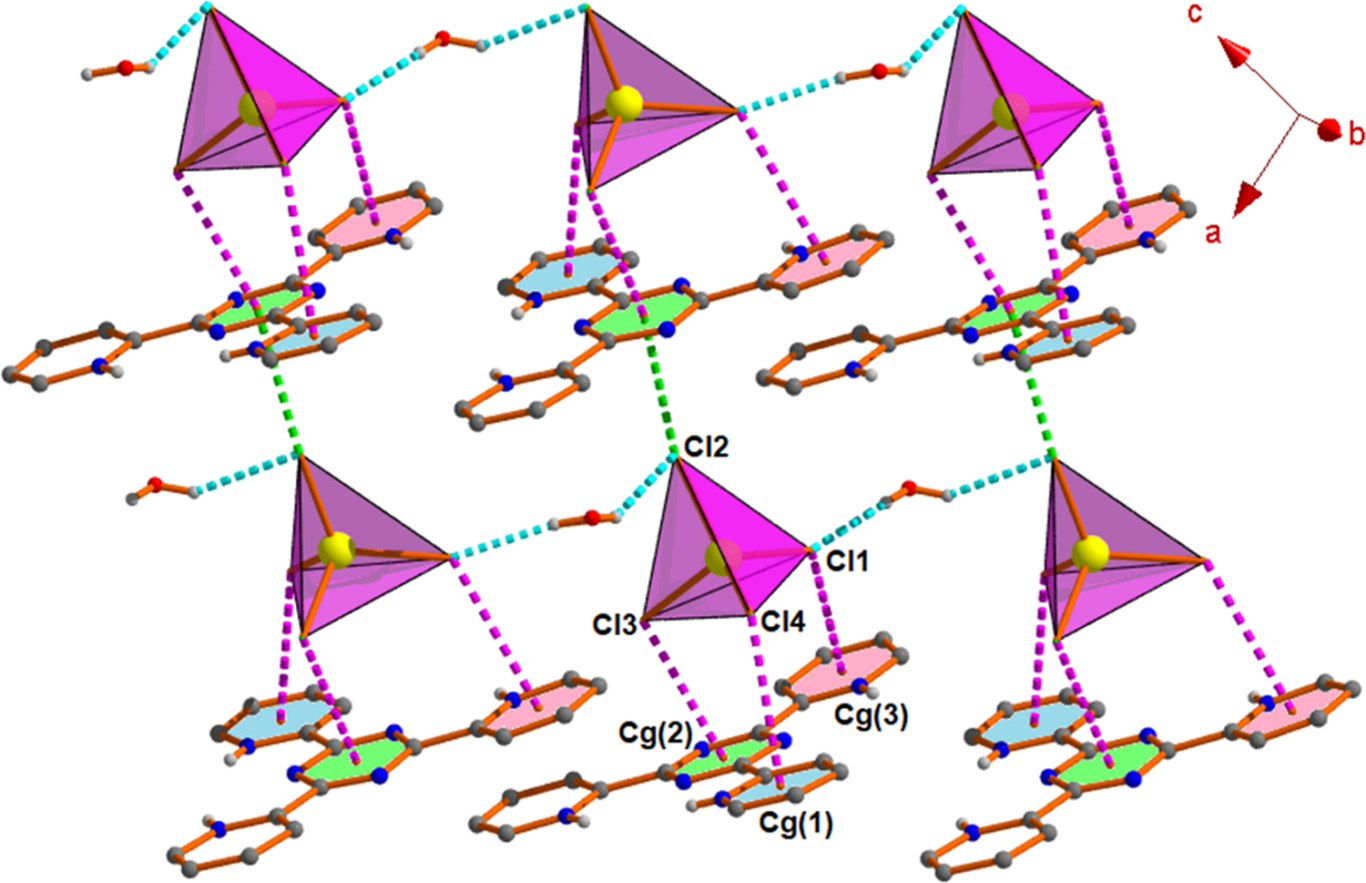
Formation of 2D architecture incorporating water–anion cluster, anion···π/π^+^ interactions in complex **2**.

In complex **2**, Cd(1)–Cl(2) and Cd(1)–Cl(3) form anion···π interactions with Cg(2), whereas Cl(1) and Cl(4) attached with Cd^2+^ interact with Cg(1) and Cg(3), respectively, in anion···π^+^ fashion [where Cg(3) is the centroid of N5C9C13C12C11C10 ring] to ensure a 1D chain (Figure 4). Now, the water–anion cluster (Figure 1b) along with these anion···π and anion···π^+^ interactions enhance the dimensionality from 1D to 2D in the *ac*-plane, as depicted in Figure 4. Here, the tripodal anion···π/π^+^interaction distances range from 3.2754(10) to 3.7185(12) Å and the corresponding angles are 98.97(3) and 163.25(3)°, respectively (Table S5).

**Figure 5:**
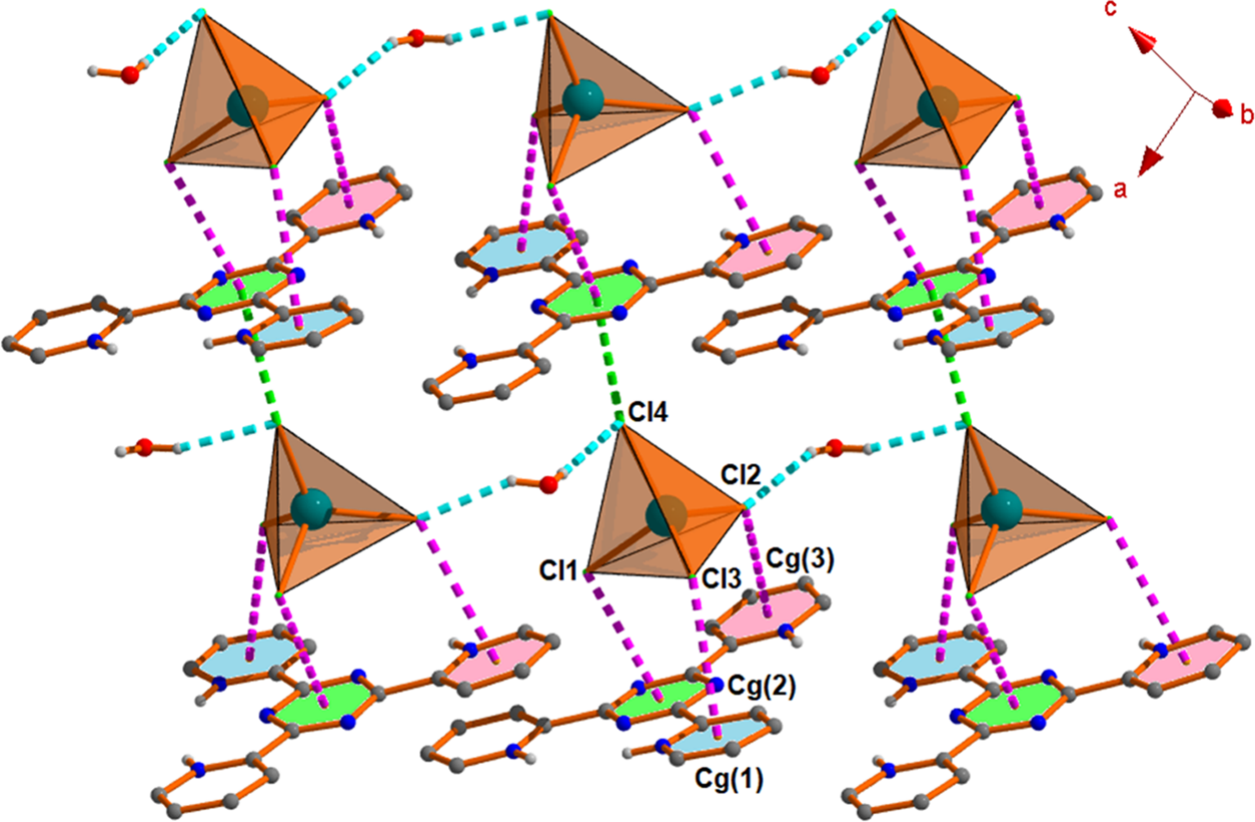
View of 2D architecture involving water–anion cluster, anion···π/π^+^ interactions in complex **3**.

Interestingly, in complex **3**, the HgCl_4_^2–^ ion interacts in a similar tripodal anion···π/π^+^ fashion with the protonated organic moieties (as that of complex **2**) to produce a 1D chain. Now, the parallel chains are interconnected through the water–anion cluster (Figure S6b) to expand the dimensionality to 2D (Figure 5). Here, Hg(1)–Cl(1) and Hg(1)–Cl(4) form anion···π interactions with Cg(2), whereas Cl(2) and Cl(3) attached with Hg^2+^ form anion···π^+^ interactions with Cg(3) and Cg(1), respectively. The tripodal anion···π/π^+^ interaction distances in this complex range from 3.251(4) to 3.691(4) Å and the corresponding angles are 98.54(8) and 161.61(11)°, respectively (Table S5).

### Hirshfeld Surface Analysis

The Hirshfeld surfaces of the three complexes (**1–3**) have been mapped over *d*_norm_, *d*_i_, *d*_e_, shape index, curvedness, and fragment patches (shown in Figures S15–S17). From the shape index surface, one can get the information about each donor–acceptor pair, whereas the curvedness surface measures how much shape successfully splits the surfaces into a set of patches. The fragment patches correspond to the environment of the nearest neighbor on the surface depending on the proximity of the adjacent molecules in the form of color patches. The surfaces were enabled transparent to visualize the molecular structures in a similar fashion for all of the complexes around which they were calculated. Furthermore, the combination of *d*_i_ and *d*_e_ in the form of a 2D fingerprint plot represents all intermolecular contacts that are involved within the structures. In the 2D fingerprint plot, the intermolecular interactions appear as individual spikes and different spikes represent their corresponding interactions. Besides, the decomposed fingerprint plots highlight particular atom pair close contacts (Figures S18–S20), and the decomposition separates the contributions of various individual interactions that overlap in the total (100%) fingerprint plot. For all of the title complexes (**1–3**), the major nonclassical hydrogen bonding interaction is observed between hydrogen and chlorine atoms. The H···Cl/Cl···H contacts contributed 42.5, 42.6, and 43.2% for each complex of the total Hirshfeld surface area of complexes **1**, **2**, and **3**, respectively. Besides, the C···Cl/Cl···C contacts (cyan color in the fingerprint plots) represent anion···π/π^+^ interactions and contribute 11.3, 9.4, and 9.8% to the total surface areas of complexes **1**, **2**, and **3**, respectively. From Figures S18–S20, one can see that the contribution of other interactions (H···N and H···O) is minimal compared to H···Cl interactions. The H···N/N···H interactions contributed 6.2, 5.1, and 5.4%, whereas the contribution of the H···O/O···H contacts was 0.8, 3.9, and 1.5% to the total Hirshfeld surface areas of the complexes (**1**–**3**), respectively.

### Optical Studies

Figures S21–S23 represent Tauc’s plot of complexes **1** (Zn^2+^-based), **2** (Cd^2+^-based), and **3** (Hg^2+^-based), respectively. Using the following Tauc’s relation, band gap energy (*E*_g_) was calculated.
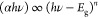
1Here, “*h*ν” represents the photon energy, “α” is the absorption coefficient, and “*n*” is a coefficient for direct band-to-band transition having a value of 1/2. By plotting (α*h*ν)^2^ vs *h*ν and extrapolating the linear line to the *x*-axis, the band gaps were calculated. The “*E*_g_” was calculated to be 3.87, 3.77, and 3.96 eV for complexes **1**, **2**, and **3**, respectively. *E*_g_ values suggest that only changing the metal ion has no such broad impact in altering the band structure of the complexes. The insets of the figures represent the ultraviolet–visible (UV–vis) absorption spectra of the complexes where no such distinct changes have been noticed for a particular complex.

### Fabrication of Device and Electrical Properties

**Figure 6:**
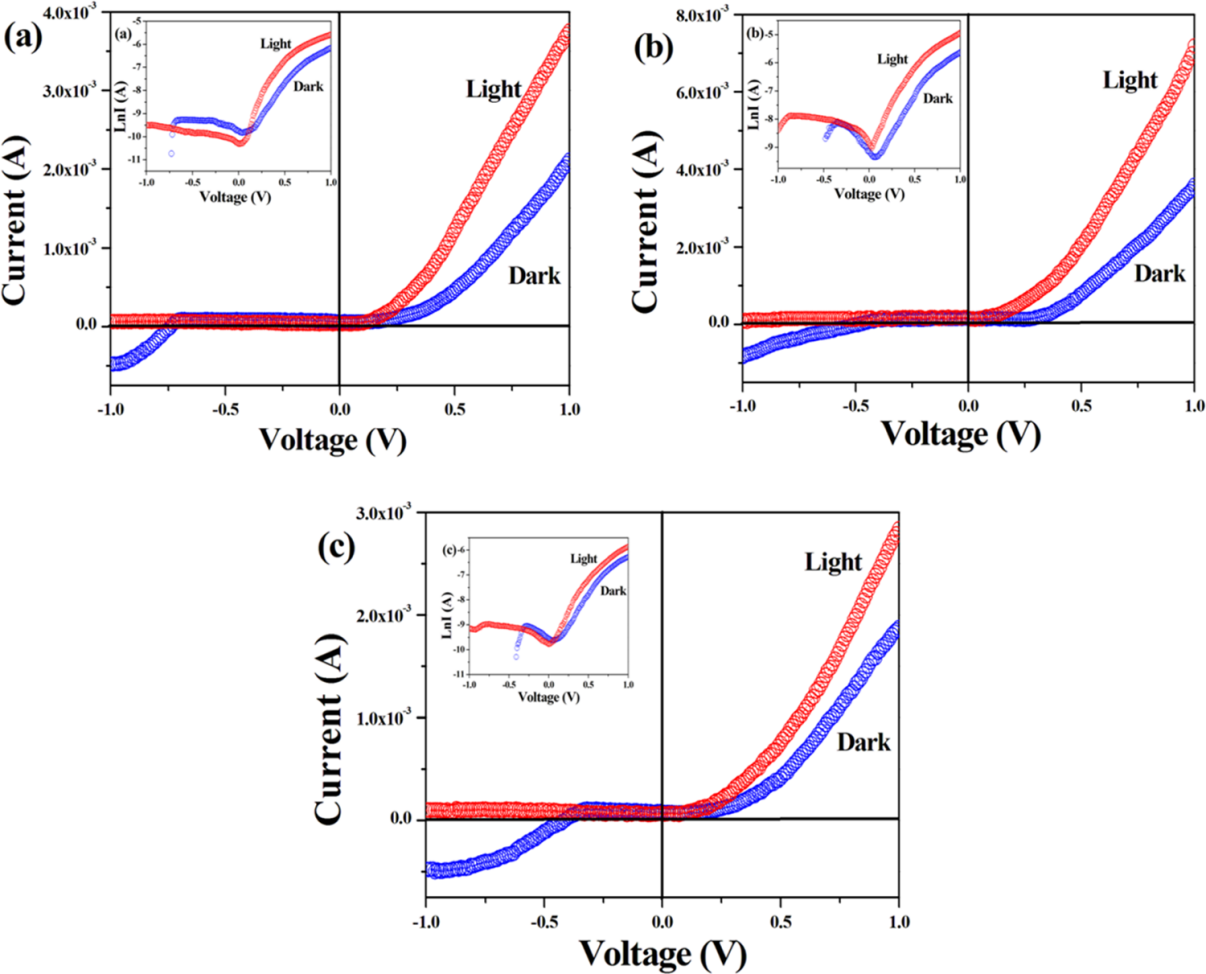
*I*–*V* characteristics of complexes **1–3** (a–c) under dark and illumination conditions (a–c); inset: ln *I*(A) vs voltage (V) plot.

To measure the electrical properties related to SBD parameters, current–voltage *I*–*V* measurement was executed. Here, two contacts were made, one was from the ITO substrate (bottom contact) and another was from the deposited part (top contact), where graphite was used as the contact metal (ITO/complex/graphite). The details of thin-film fabrication from the precursor complexes are given in the Supporting Information. Experiments were performed with a highly sophisticated *I*–*V* analyzer (Keithley 4200) under dark and light conditions in the voltage range of ±1 V. Figure 6a–c shows *I*–*V* characteristics of complexes **1**, **2**, and **3**, respectively. It is obvious that the *I*–*V* nature of complex **2** displays enhanced nonlinear rectifying performance than complexes **1** and **3**. The nonlinearity of the *I*–*V* curve specifies that for all of the complexes (**1–3**), the conduction mechanism is non-ohmic, and rectifying nature indicates the Schottky barrier diode (SBD) characteristics of the devices. The conductivity of the complexes at room temperature under dark and light conditions has been measured. In the presence of light, the conductivity increased from 7.20 to 15.64 S/m for complex **1**, 10.72 to 22.35 S/m for complex **2**, and 7.89 to 13.5 S/m for complex **3**. In all of the cases, conductivity was found to be increased in the presence of light and the higher conductivity of complex **2** than those of **1** and **3** indicates the significant photoresponse behavior of device 2. As the complexes show a wide band gap, thermionic emission theory could be applied to justify the electrical properties.^36^ All of the *I*–*V*’s and their relative parameters are quantitatively examined and authenticated by Cheung’s equation.^37^
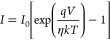
2where *I*_0_ is the saturation current was calculated from the intercept of ln(*I*) at *V* = 0 and can be simplified as follows
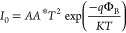
3where the terms *q*, *K*, *T*, *V*, *A*, A*, and η stand for the electronic charge, Boltzmann constant, temperature (in kelvin), forward bias voltage, effective diode area, Richardson constant, and ideality factor, respectively. In this case, the effective diode area is maintained as 8.0 × 10^–2^ cm^2^, and for all the SBDs, the Richardson constant is considered as 32 A K^–2^ cm^–2^. Now, forward *I*–*V* characteristics can also be expressed by the following equation.
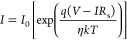
4Here, *IR*_s_ symbolizes a drop in voltage diagonally the series resistance and it is determined by the following equation.
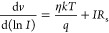
5Equation 5 can be represented another way as a function of *I.*
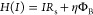
6Again, *H*(*I*) can also be written using the following equation.
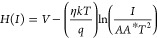
7Figure S24 represents the d*V*/d(ln *I*) vs *I* plot for all of the devices under dark and light conditions. The slope of this plot indicates series resistance (*R*_s_), and from the intercept, the ideality factor (η) was computed. The *R*_s_ values of the devices (**1**–**3**) were found to be 7.76 and 2.76, 6.78 and 5.63, 0.256 and 5.33 under dark and light conditions, respectively. The low “*R*_s_” value for device **2** indicates its easy passage of electron tunneling.

The “η” values of the devices were found to be 4.1, 4.0, and 4.13 under dark conditions. Under the illumination of light, the η values were reduced to 1.48, 1.39, and 1.7 for respective devices. From the η values, it is clear that the metal–semiconductor (MS) junctions of the SBDs are not exactly perfect. The factors responsible for this deviation are the difference in interfacial states, inhomogeneities in barrier height potential, and high series resistance at the MS junction. Here, complex **2** is comparatively ideal for SBD.

Figure S25 represents the *H*(*I*) vs *I* plot for all devices under dark and light conditions. The *H*(*I*) vs *I* plot gives a straight line where the intercept along the *y-*axis becomes equal to ηϕ_B_, and the slope gives *R*_s_. Now, by using the measured η values, potential barrier heights (Φ_B_) for respective devices were calculated. All of the device-related parameters including potential height (ϕ_B_), series resistance (*R*_s_), and ideality factor (η) are listed in Tables S6 and S7.

**Figure 7:**
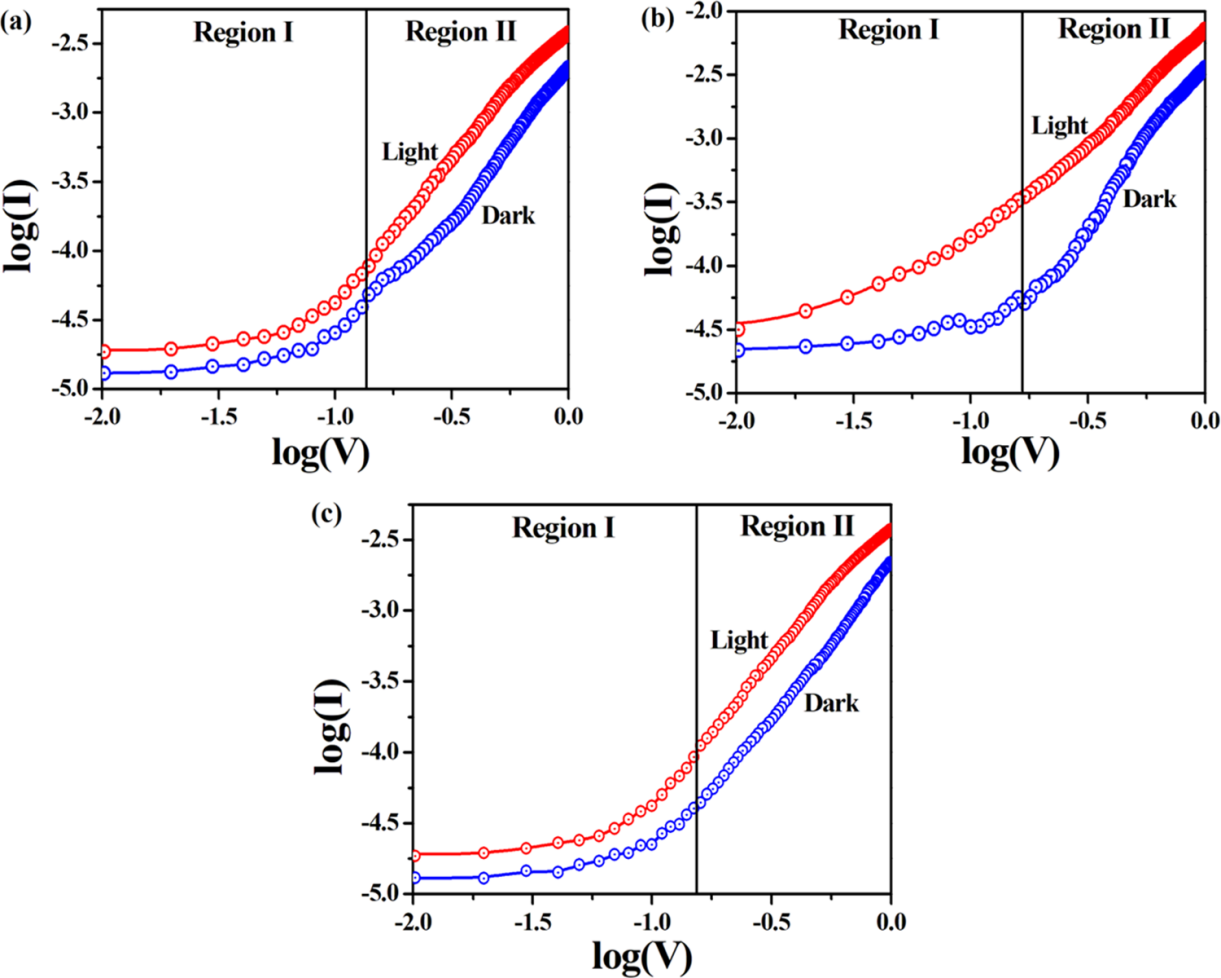
Log(*I*) vs log(*V*) plot of complexes **1–3** (a–c) under dark and illumination conditions, where *I* and *V* represents forward current and voltage, respectively.

To quantify a detailed study of charge transport phenomena at the interface of the MS junction, space-charge-limited current (SCLC) theory is introduced. Using this theorem, several SBD parameters like carrier mobility (μ_eff_), carrier concentration (*N*), diffusion coefficient (*D*), diffusion length (*L*_D_), and transient response time (τ) have been calculated. To acquire a significant outcome with respect to the carrier transport phenomenon at the interface of the MS junction, log(*I*) vs log(*V*) plots were drawn for the forward bias (Figure 7). This plot is compared with the power law (*I* ∝ *V*^m^) to locate the charge transfer zone in the MS junction. The slope value (*m*) fences the boundary in between ohmic and SCLC regions.

For the ohmic nature, the *m* value will be in the range of less than or equal to 1. Further greater than 1 or equal to 2 (*m* ≥ 2) indicates a space-charge-limited current (SCLC) region.^38^Figure 7a–c represents log(*I*) vs log(*V*) plots of devices **1**, **2**, and **3**, respectively, under dark and illumination conditions. The log(*I*)–log(*V*) plot illustrates two distinct linear regions (I and II) of different slopes, hence concluding that two different conduction mechanisms are being operated in the MS junction. At low potential region I, current becomes proportional to the applied voltage (*I* ∝ *V*), and hence, the device exhibits an ohmic nature in this zone. Region I attributes thermionic emission where bulk-generated electrons are responsible to produce current than the injected free carriers from the metal.^39,40^ At a higher potential, the log(*I*)–log(*V*) plot obeys the simple power law (*I* ∝ *V*^m^), and it is separated as region II. In region II, the current becomes proportional to the square of the applied voltage (*I* ∝ *V*^2^), specifying that the current is run by space-charge-limited current (SCLC) obtained from the discrete trapping level.^41,42^

From the *I* vs *V*^2^ graph (Figure S26) and using the Mott–Gurney equation,^43^ carrier mobility (μ_eff_) was evaluated.
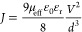
8where the terms “*J*”, “ε_0_”, “ε_r_”, and “*d*” stand for the current density, permittivity of free space, relative dielectric constant of complexes, and film thickness. Thicknesses of the films were about 2 μm for all of the devices, which have been measured by a surface profilometer (Bruker Contour GT; noncontacting mode). Before this measurement, dielectric studies were made for all of the complexes. Dielectric constants for all of the complexes have been calculated from the plots of capacitance vs frequency (Figure S27). Capacitance was measured by varying the frequency between 1 and 10 kHz, under a constant bias of 1 V. By using the conventional equation,^44^ relative dielectric constants for complexes **1**, **2**, and **3** were measured and found 6.23, 2.27, and 4.13, respectively. The relatively lower dielectric constant of complex **2** is an indication of its potential use as an optoelectronic device to reduce power loss.

Transit time (τ) of the charge carriers is another important parameter to investigate charge transport across the junction. From the slope of the forward *I*–*V* curve (Figure 6), τ was calculated using the following equation.^45^
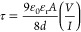
9The details of these parameters are presented in Table S7. The higher effective mobility value of device **2** confirms fast charge transport of the material through the MS junction compared to **1** and **3**. The mobility enhancement of the carrier due to light illumination indicates the good photoresponsivity of the device. The results demonstrate that the charge transport property of device **2** (made from complex **2**) is better than those of devices **1** and **3**. All of these SBD-related parameters imply the ultimate supremacy of complex **2**.

### DFT Studies

#### Theoretical Study for Noncovalent Interactions

**Figure 8:**
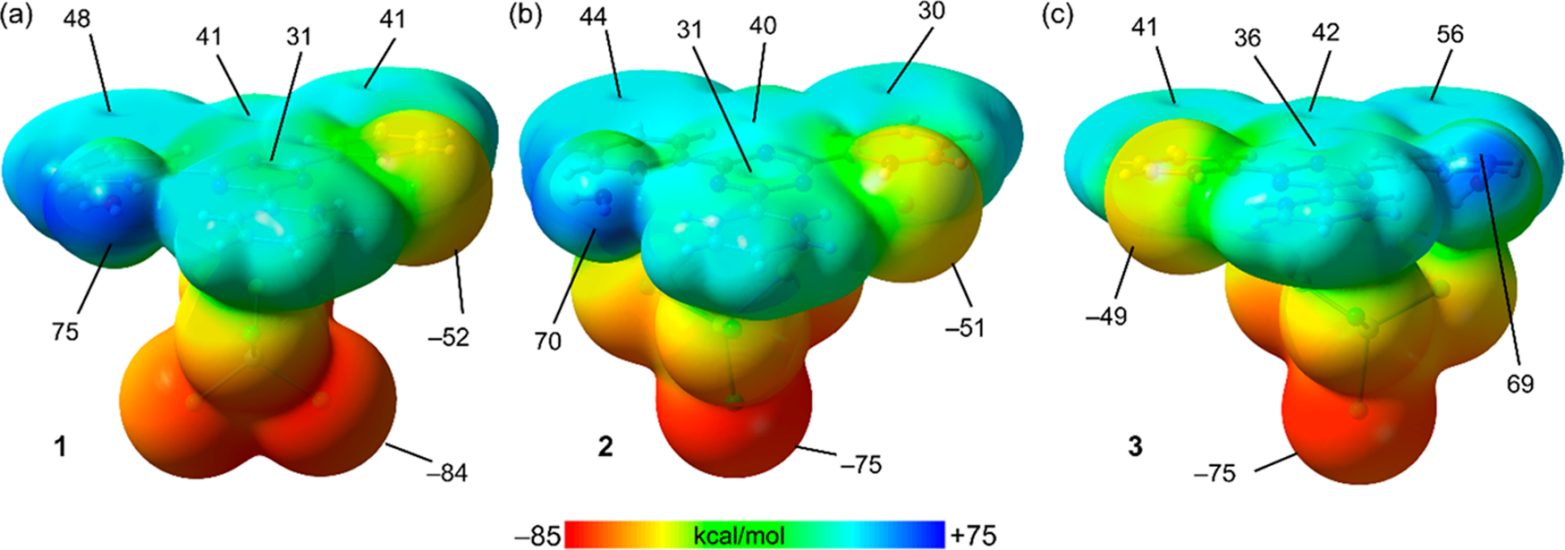
MEP surfaces of complexes **1**–**3** (a–c) with an indication of the values at some points of the surface in kcal/mol. Plots generated with the GaussView 6.0 program.^46^

As detailed above, all complexes reported herein exhibit structure-directing anion−π and hydrogen bonding interactions between the protonated organic ligand, counterions, and water molecules. The DFT study is devoted to analyze and compare the interactions in complexes **1**–**3**. First, we have computed the MEP surfaces of a neutral model of each complex that consists of tricationicH_3_tptz^3+^ with the hydrogen-bonded Cl^–^ and one anion−π bonded [MCl_4_]^2–^ as counterions and the hydrogen-bonded water molecule. The geometries used for the MEP calculations were extracted directly from the crystallographic coordinates, since we are interested in evaluating the interactions, as they stand in the solid state. The MEP surfaces for these models of complexes **1**–**3** are represented in Figure 8, evidencing that the MEP maxima are located at the H-atoms of the water molecule, ranging from 69 to 75 kcal/mol.

**Figure 9:**
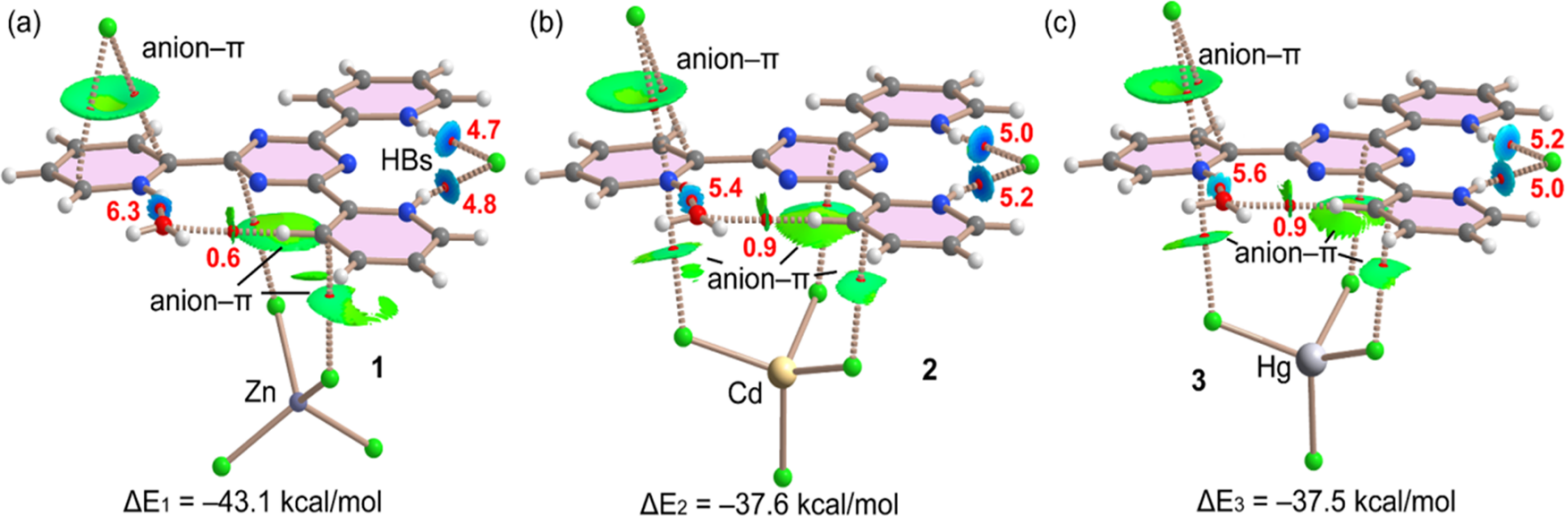
Combined QTAIM (bond CPs in red and ring CPs in yellow) and NCI plot (RDG = 0.5, color scale −0.03 au. ≤ sign(λ_2_)ρ ≤ 0.03 au., ρ cutoff = 0.04 a.u.) analysis of the ternary anion−π/HBs assemblies observed in complexes **1** (a), **2** (b), and **3** (c). The dissociation energies of the H-bonds are indicated using a red font close to the bond CPs. The plot is generated with the AIMAll program.

The MEP values over the aromatic rings of the ligand are also large and positive, ranging from 30 to 56 kcal/mol, thus revealing the existence of an extended π–acidic surface. The MEP minima are located at the [MCl_4_]^2–^ counterions, ranging from −75 to −84 kcal/mol. The MEP is also large and negative at the hydrogen-bonded Cl atoms, ranging from −49 to −52 kcal/mol. We have also analyzed the energies of the anion−π and anion−π^+^ interactions using DFT calculations and characterized the interactions using a combination of the QTAIM and NCI plot analyses, since they are very convenient to reveal noncovalent interactions in real space. We have first analyzed the energies of the neutral assemblies used to construct the MEPs shown in Figure 8 with the Cl^–^ anion (anion−π^+^). That is, the energies summarized in Figure 9 were calculated as dimers, where one monomer is the chloride anion and the other one is the [(H_3_tptz)(Cl)(MCl_4_)(H_2_O)] assembly. The anion−π energies are very large and negative due to the large electrostatic attraction between the anion and the protonated pyridine ring. The stronger energy is found for complex **1** (Δ*E*_1_ = −43.1 kcal/mol), while for complexes **2** and **3**, it is almost identical, likely due to the similar orientation of the [MCl_4_]^2–^ anion at the opposite side of the Cl···π interaction. The combined QTAIM/NCI plot analyses for the assemblies of [(H_3_tptz)(Cl)(MCl_4_)(H_2_O)] interacting with Cl^–^ are represented in Figure 9, showing that each Cl^–^ anion is connected to the aromatic ligand via two bond critical points (CPs, red spheres) and bond paths (dashed bonds), thus confirming the existence of the anion−π^+^ interactions. The anion−π nature of the interaction is further confirmed by the size and shape of the reduced density gradient (RDG) isosurfaces, embracing the region between the π-system and the chlorine atom. We have also computed the energies of the hydrogen bonds by using the values of the potential energy density (*V*_r_) at the bond CPs. It can be observed that in all complexes, the chloride is connected to the NH groups via bond CPs and bond paths and is further characterized by small blue isosurfaces.

The water molecule is also connected to one NH bond via a bond CP, bond path, and blue isosurface. Moreover, in all of the complexes (**1**–**3**), a secondary CH···O (water) interaction is also established. The energies of the hydrogen bonds are indicated in red adjacent to the bond CPs. In complexes **2** and **3**, the hydrogen bond energies are almost identical, with a total of 10.2 kcal/mol for both NH···Cl hydrogen bonds and 5.4 and 5.6 kcal/mol for the NH···O(water). For complex **1**, the NH···Cl hydrogen bonds are slightly weaker (9.5 kcal/mol in total) and the NH···O(water) hydrogen bond is stronger (6.3 kcal/mol). The secondary CH···O(water) contacts are significantly weaker (0.6 kcal/mol for **1** and 0.9 kcal/mol for **2** and **3**) in line with the small and green NCI plot isosurface.

**Figure 10:**
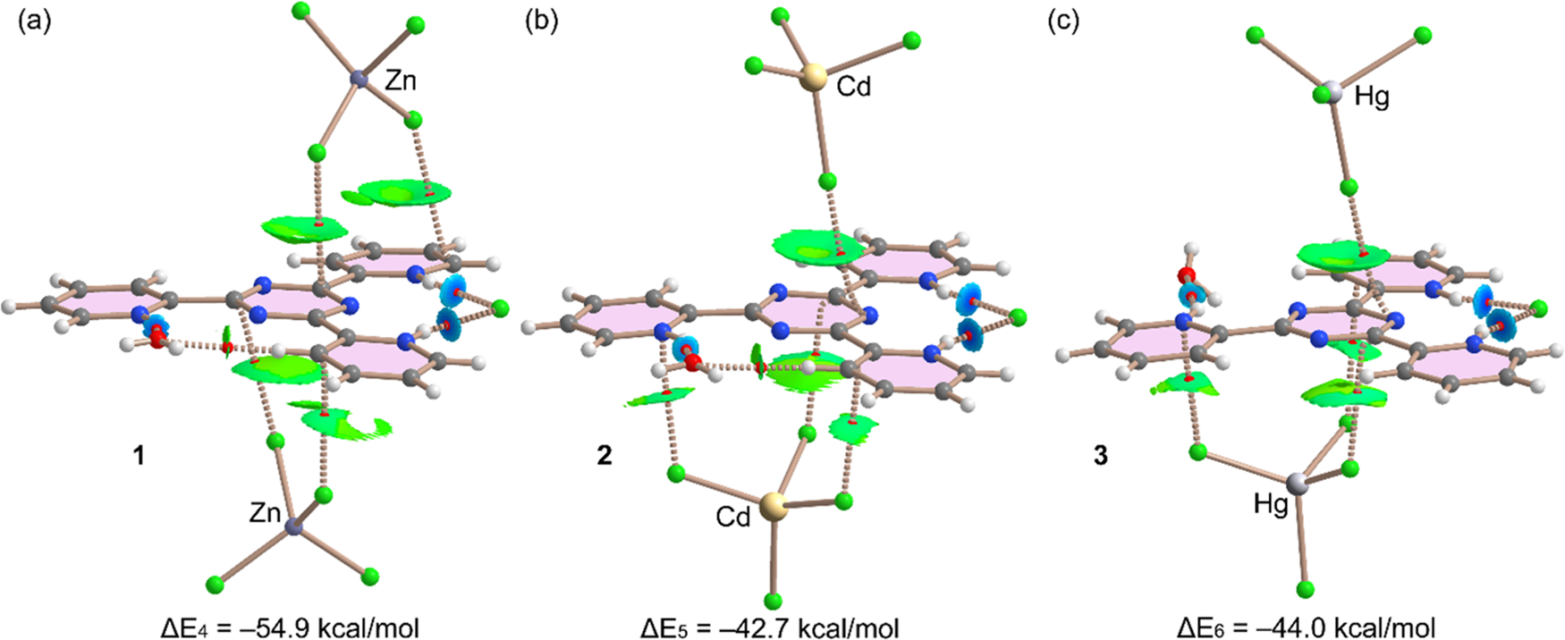
Combined QTAIM (bond CPs in red and ring CPs in yellow) and NCI plot (RDG = 0.5, color scale −0.03 a.u. ≤ sign(λ_2_)ρ ≤ 0.03 a.u., ρ cutoff = 0.04 a.u.) analysis of the anion−π/HBs assemblies observed in complexes **1** (a), **2** (b), and **3** (c). The plot is generated with the AIMAll program.

We have also evaluated the anion−π energies for the assemblies of [(H_3_tptz)(Cl)(MCl_4_)(H_2_O)] with the [MCl_4_]^2–^ anions, as detailed in Figure 10. The strongest interaction is observed again for complex **1** (Δ*E*_4_ = −54.9 kcal/mol), while for complexes **2** and **3**, the energies are Δ*E*_4_ = −42.7 kcal/mol and Δ*E*_6_ = −44.0 kcal/mol, respectively.

This is likely due to the different binding modes observed in **1**, where the two Cl atoms are pointing to the acidic π-surface of the ligand, while in complexes **2** and **3**, only one Cl atom of the anion points to the central ring. In the latter complexes, only one bond CP and bond path connect the anion to the N atom of the ligand. However, the shape and extension of the NCI plot isosurface confirm the anion−π nature of this interaction, though the QTAIM analysis only reveals a single Cl···N contact. In the case of complex **1**, two bond CPs and bond paths connect the anion to the ligand, one to the central triazine ring, and the other one to one protonated pyridine ring. The interaction energies obtained for the anion−π complexes involving the [MCl_4_]^2–^ anions (Figure 10) are stronger than those involving the Cl^–^ anion (Figure 9) due to their monoanionic nature and consequently weaker electrostatic attraction.

### Theoretical Study for Photophysical Properties

**Figure 11:**
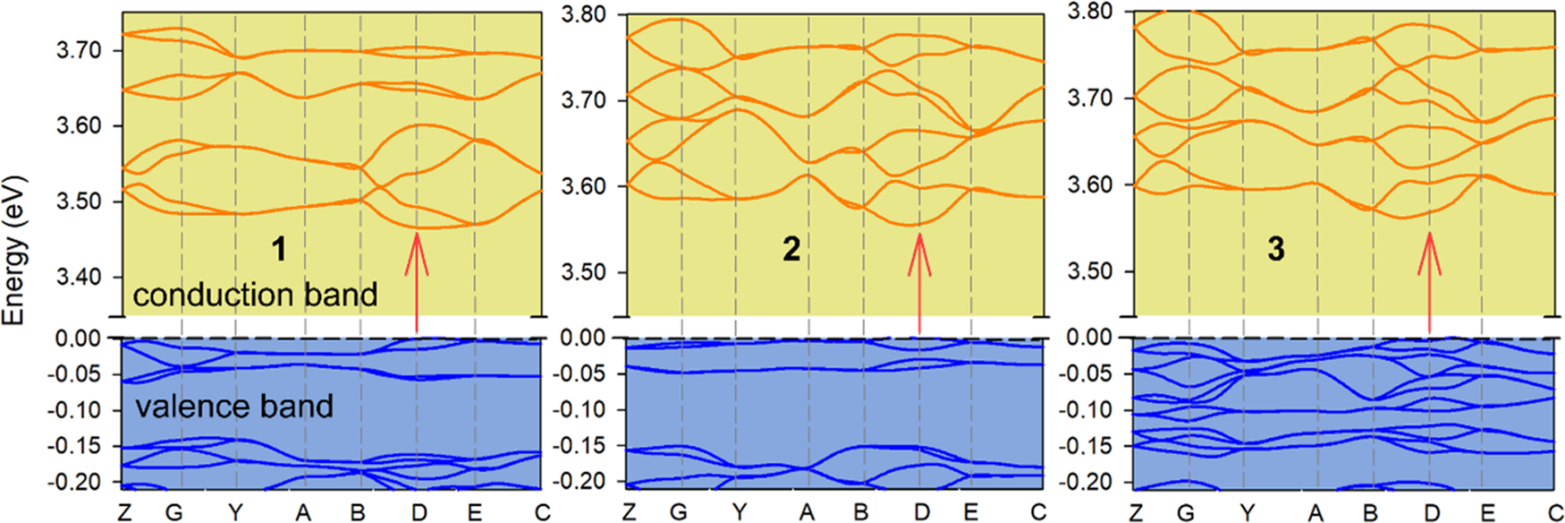
Electronic band structures of the ground state for **1**, **2**, and **3** crystal structures. Points of high symmetry in the first Brillouin zone are labeled as follows: *Z* = (0, 0, 0.5); *G* = (0, 0, 0); *Y* = (0, 0.5, 0); *A* = (−0.5, 0.5, 0); *B* = (−0.5, 0, 0); *D* = (−0,5, 0, 0.5); *E* = (−0.5, 0.5, 0.5); and *C* = (0, 0.5, 0.5). The red arrow marks the band gap.

The solid-state structures of the **1**, **2**, and **3** crystals were modeled using TD-DFT methodology and choosing the experimental crystal lattices as a starting point for optimizing the atomic position. Standard band theory was used to determine the adjustment degree between the experimental and theoretical data. Figure 11 shows the band diagram between the crystals in the study. All systems present direct band gap behavior at point D of the Brillouin zone and around 3.50 eV. This is in reasonable agreement with the experimental values that range from 3.77 to 3.96 eV, thus evidencing that the TD-DFT method slightly underestimates the band gap values. Nevertheless, the theoretical calculations confirm the semiconductor type and low influence of the metal atom.

**Figure 12:**
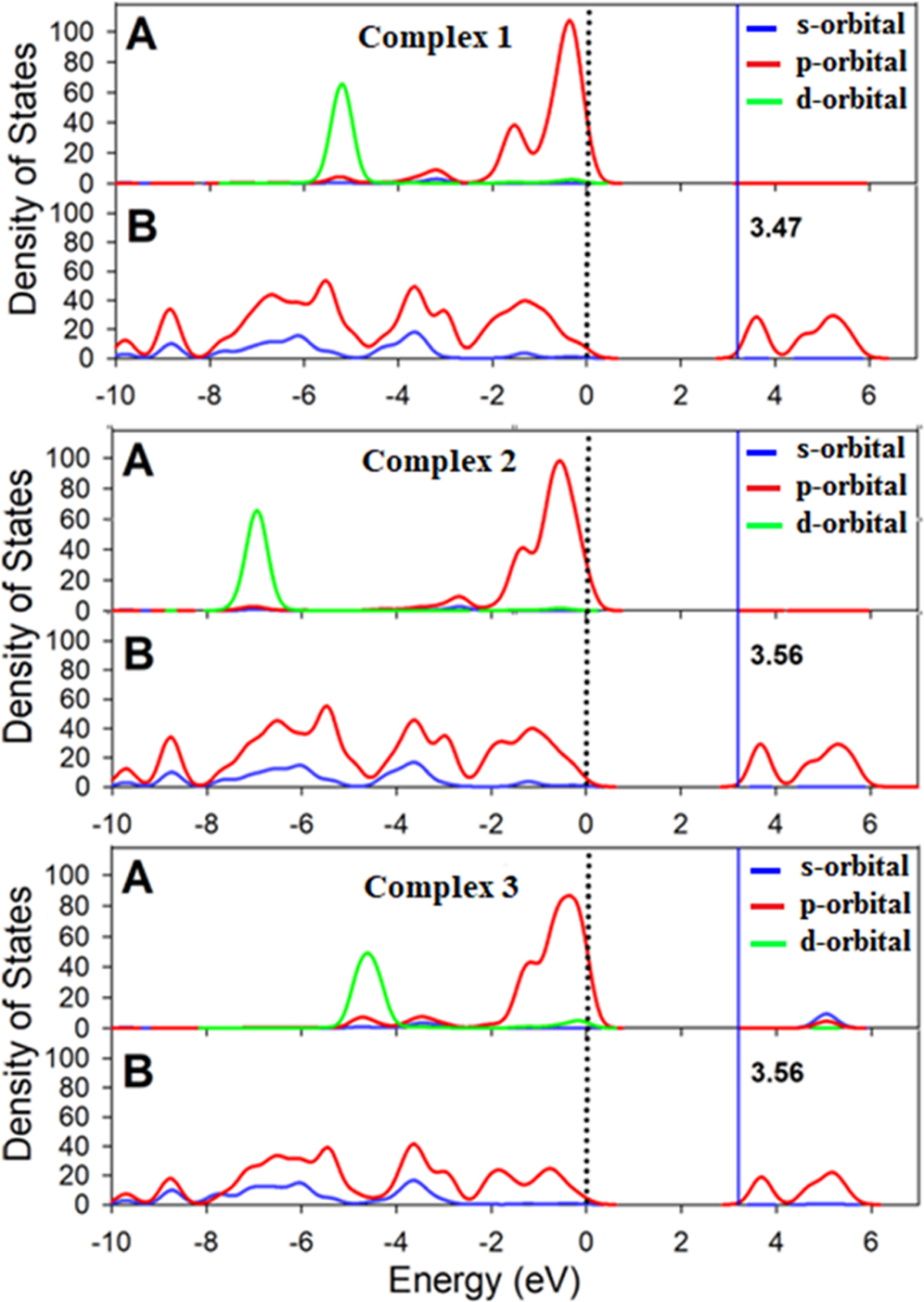
Calculated total and partial density of states of **1**, **2**, and **3** crystal cells. Panels show: (**1**) (A) [ZnCl_4_]^2–^ and Cl^–^ anions, (B) tptz ligands; (**2**) (A) [CdCl_4_]^2–^ and Cl^–^ anions, (B) tptz ligands; (**3**) (A) [HgCl_4_]^2–^ and Cl^–^ anions, (B) tptz ligands. The lines represent the s-orbital character (blue), p-orbital character (red), and d-orbital character (green) of the atoms in the crystal.

Figure 12 shows the partial density of states for the three complexes. The plots represent the contribution of [MCl_4_]^2–^ (M = Zn, Cd, Hg) and chloride anions and the tptz ligand separately. It is revealed that the p-character of the anions mainly dominates the valence band, and the p-character of the tptz ligands mainly dominates the conduction bands. Interestingly, the d-orbitals of the metal centers do not participate either in the conduction bands or in the valence bands, in line with the negligible effect of the group 12 element on the band gap.

**Figure 13:**
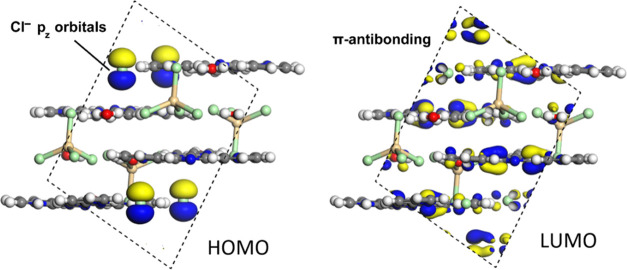
Representation of HOMO–LUMO of complex **2**. The orbitals are represented using the 0.025 a.u. isovalue.

**Figure 14:**
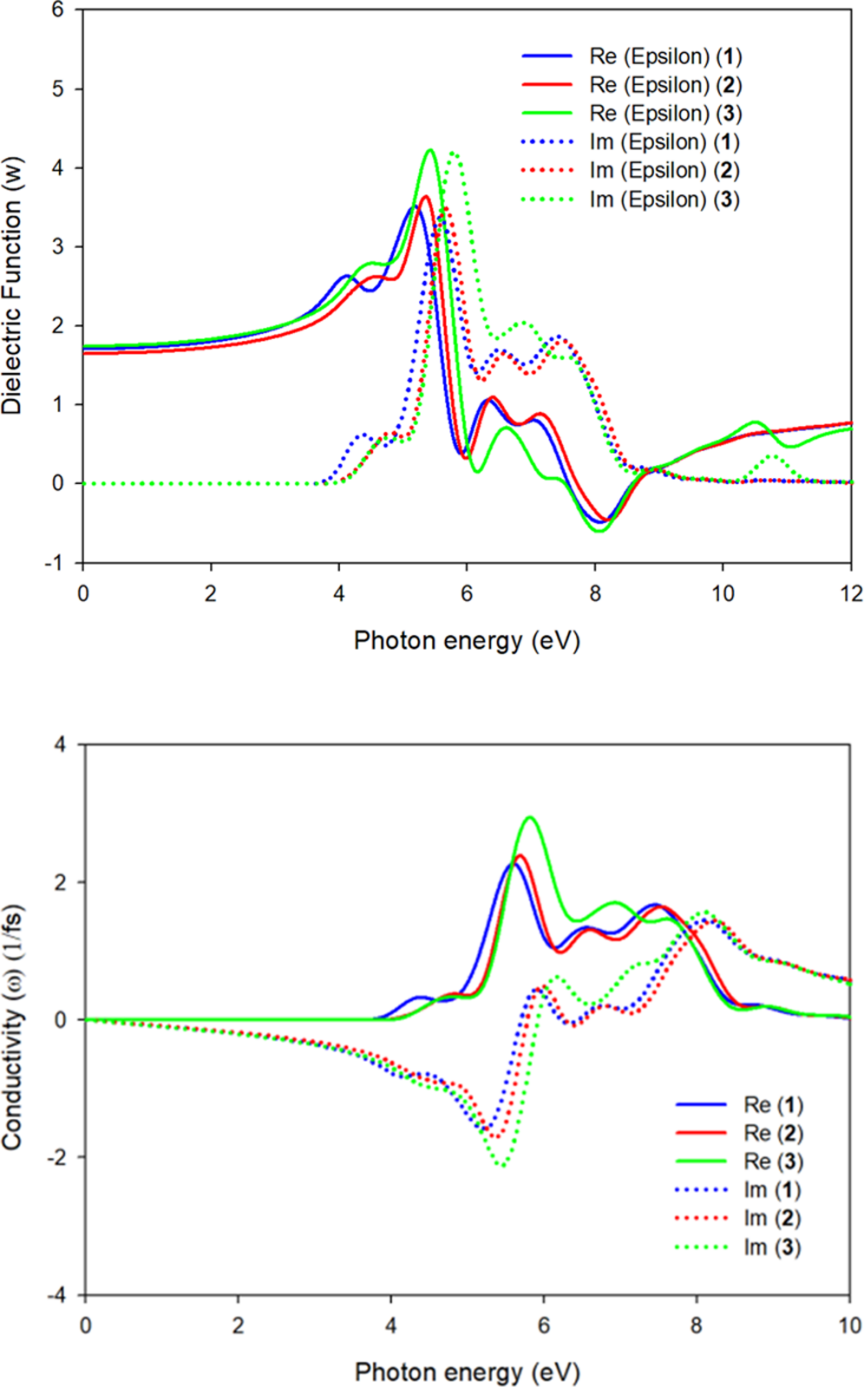
Upper panel shows a plot of real (solid line) and imaginary (point lines) parts of the dielectric function vs the photon energy of **1** (blue line), **2** (red line), and **3** (green line) crystals. The lower panel shows a plot of real (solid lines) and imaginary (points lines) parts of optical conductivity vs the photon energy of **1** (blue line), **2** (red line), and **3** (green line) crystals.

This fact is also verified by calculating the frontier molecular orbitals in all of the systems that show the total absence of density at the [MCl_4_]^2–^ anions in both frontier orbitals. Figure 13 shows the highest occupied molecular orbital (HOMO) and lowest unoccupied molecular orbital (LUMO) of complex **2** as representative crystal structures, since these are almost identical in the three complexes. The HOMO plot in Figure 13 shows the contribution of the p-orbital at the chloride anion, while the LUMO is a π-antibonding orbital localized at the tptz ligand. Theoretically, it is possible to know the frequency dependence of an incident photon in a material with the calculation of the dielectric function ε(ω) (see Figure 14).^47−52^ We have chosen a photon energy range of 0–12 eV to calculate the optical response using the band structure. Figure 14 shows that complexes **1**–**3** have similar dielectric constant diagrams concerning the intensity of their maxima, in agreement with the similar experimental behavior of complexes **1**–**3**. We have also measured the optical conductivity of the material (σ(ω)), since it is helpful to analyze how the conductivity of the material changes upon illumination. Moreover, the photoconductivity and hence electrical conductivity of materials increase because of absorbing photons.^47−52^

As a rough approximation, it is possible to relate the optical conductivity of the material with the electrical conductivity measured experimentally, verifying whether any change in its electronic configurations occurs when the material absorbs a photon.^53−56^ These facts can be related to the increased conductivity of these materials when illuminated. However, the results shown in Figure 14 do not explain the fact that complex **2** is superior to **1** and **3** in terms of optical properties. A likely explanation for this is likely related to the fabrication of the device like differences in the junctions rather than differences in the photophysical properties of the compounds.

Recently, a number of efforts have been explored to induce conductivity via through-bond, extended conjugation, and through-space involving weak interaction strategies.^57−59^ In this work, the charge transportation occurs through the combination of these three strategies. The extended conjugation in the protonated organic moiety (H_3_tptz^3+^) and metal-induced charge transfer along with other weak interactions explains the higher conductivity in the title complexes (**1–3**). Here, such striking differences in device properties among the complexes can majorly be attributed to two factors: (i) the increased electron density of the protonated organic moiety (H_3_tptz^3+^) by partial electron transfer from MCl_4_^2–^ and Cl^–^ anions involved in anion−π/ anion−π^+^ interactions and (ii) the possibility of effective charge transport through the strong hydrogen bonding interactions between the MCl_4_^2–^ anions and noncoordinated water molecules.

The electrical conductivity depends not only on the ease of electron transportation through several intermolecular (covalent and noncovalent interactions) interactions but also on the band gap between the valence and conduction bands of the materials. As the electronegativity values of Zn and Cd (in Pauling scale 1.65 and 1.69, respectively) are more or less similar, it was expected that both complexes exhibit the same electrical conductance properties, but the experimental result reveals that complex **2** is somewhat higher conducting than complex **1**. This may be due to the combined effect of a lower band gap (3.77 eV) and better electron hopping through the 2D water–anion cluster (that is absent in complex **1**) of complex **2**. In the case of complex **3**, though it adopts a similar interaction network (including 2D water–anion cluster) as complex **2**, it interestingly exhibits relatively lower electrical conductivity than other complexes. This observation can be explained by the higher band gap (3.96 eV) arising from the exceptionally higher electronegativity of Hg (2.00 in the Pauling scale because of both “*f*” contraction and relativistic effects) in complex **3**.

## Conclusions

In summary, a series of tetrachlorometallates including Zn(II) (complex **1**), Cd(II) (complex **2**), and Hg(II) (complex **3**) have been synthesized with the help of a triply protonated tptz (H_3_tptz^3+^) ligand and characterized by single-crystal X-ray analysis. The crystallographic analysis reveals that anion···π, anion···π^+^, and several hydrogen bonding interactions stabilize the self-assembled structures, which in turn are responsible for the expansion of dimensionality for all of the complexes. The Hirshfeld surfaces and the related 2D fingerprint plots of **1–3** structures facilitate a comparison of hydrogen bonding interactions significantly. Besides, we have been able to fabricate SBD devices with the d^10^ metal series. The devices made from complexes **1**, **2**, and **3** exhibit non-ohmic rectifying nature under dark and illumination conditions. The measured device parameters, e.g., conductivity, effective mobility, transit time, carrier concentration, diffusion coefficient, diffusion length, and ideality factor, authenticate that complex **2** made with Cd^2+^ is more ideal as an SBD than complex **1** and **3**.

A DFT study has been used to disclose the interplay among these interactions and compare them toward the stability of the complexes. Furthermore, the interactions were also rationalized and analyzed by using MEP surfaces as well as by the combined QTAIM/NCI plots. The electrical properties of all of the complexes were analyzed theoretically. But the superiority of complex **2** as an SBD was critically explained by the band gap, differences in electronegativities of the central metal atoms, and better supramolecular interactions in it. We envision that the light-harvesting nature of these complexes could be exploited for optical as well as photovoltaic applications, which are the main focus of our ongoing investigations.

## Supporting Information Available

Synthetic scheme; physical measurements; X-ray crystallographic analysis; table for crystal parameters; geometrical parameters for hydrogen bonding and, anion···π/π^+^ interactions; FT-IR spectra; asymmetric units of the complexes; PXRD pattern analysis; thermogravimetric analysis; supramolecular assemblies; Hirshfeld surface analysis; fingerprint plots; optical characterization; device fabrication; device parameters; and theoretical methods (PDF)
