# Supplementary material for: Crystallographic Aspects, Photophysical Properties, and Theoretical Survey of Tetrachlorometallates of Group 12 Metals [Zn(II), Cd(II), and Hg(II)] with a Triply Protonated 2,4,6-Tris(2-pyridyl)-1,3,5-triazine Ligand
Source: Inorg Chem. 2023 May 2;62(19):7220–34. doi: 10.1021/acs.inorgchem.2c04521 (PMC10189735; doi:10.1021/acs.inorgchem.2c04521)
Supplement: Supplementary file 1 — ic2c04521_si_001.pdf [file ic2c04521_si_001.pdf]

SUPPORTING INFORMATIONS

Crystallographic Aspects, Photophysical Properties and Theoretical Survey of Tetrachlorometallates of Group 12 Metals [Zn(II), Cd(II) and Hg(II)] with Triply Protonated 2,4,6 Tris-(2-pyridyl)-1,3,5-triazine Ligand

Samit Pramanik[a], Sumanta Jana[a], Kinsuk Das[b], Sudipta Pathak[c], Joaquin Ortega-Castro[d], Antonio Frontera[d,*] Subrata Mukhopadhyay[a,*]

[a]Department of Chemistry, Jadavpur University, Kolkata 700032, India

[b]Department of Chemistry, Chandernagore College, Hooghly, West Bengal 712136, India

[c]Department of Chemistry, Haldia Government College, Debhog, Purba Medinipur, West Bengal 721657, India

[d]Department of Chemistry, Universitat de les Illes Balears, Crta. de Valldemossa km 7.5, 07122 Palma de Mallorca (Baleares), SPAIN.

Antonio Frontera - Email: toni.frontera@uib.es.

Subrata Mukhopadhyay - Email: ju_subrata@yahoo.co.in

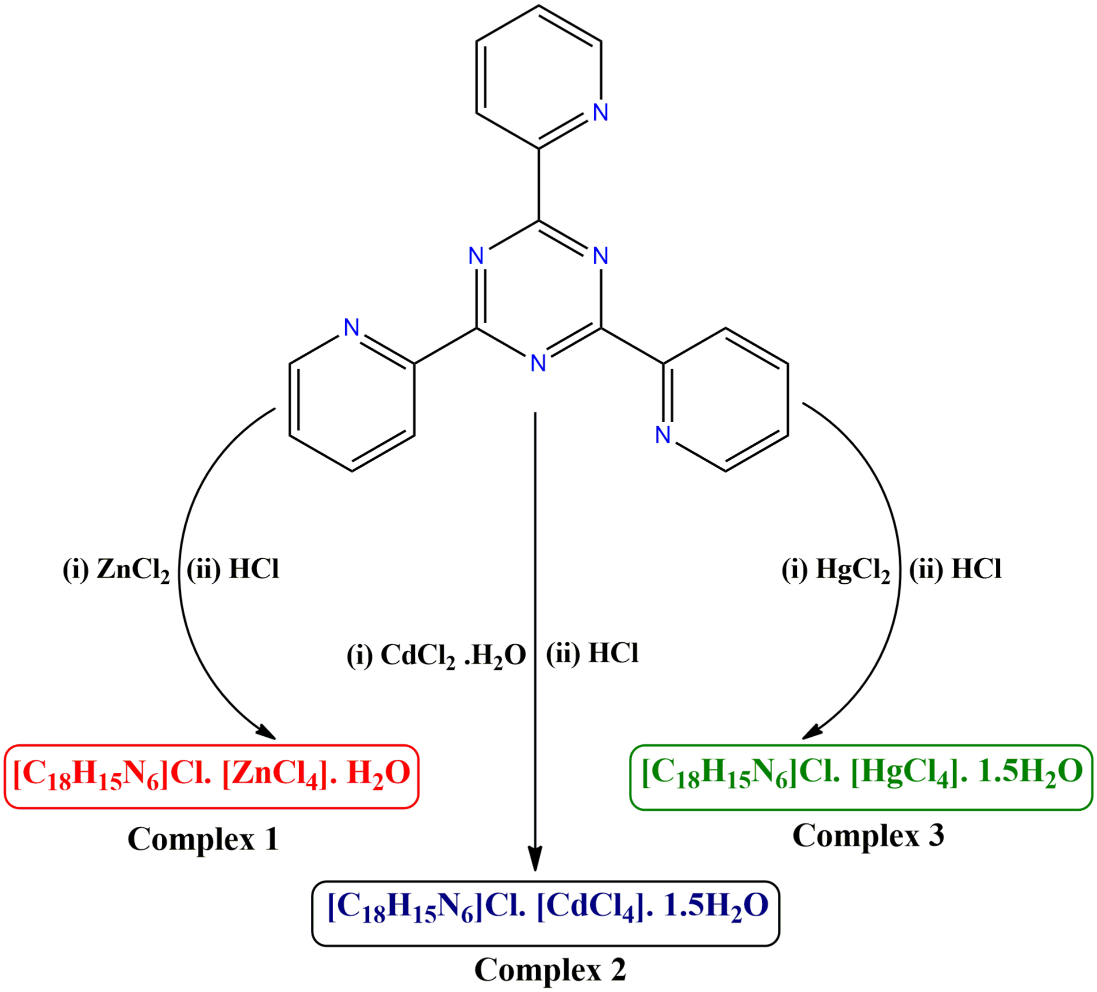

Scheme S1. Schematic Representation of the Synthesis of the Title Complexes.

Materials and Physical Measurements

All starting materials were of analytically pure grade and 2,4,6-tris(2-pyridyl)-1,3,5-triazine was purchased from commercial source(Sigma-Aldrich) and used without further purification. All the reactions were carried out under aerobic conditions and in aqueous medium. Freshly boiled, doubly distilled water was used during all the experiments.

Elemental analyses (C, H and N) were performed using a PerkinElmer 2400 Series-II CHN analyzer. Fourier transform-infrared (FT-IR) spectra of complexes **1**, **2** and **3** were recorded

using a PerkinElmer LX-1 FTIR spectrophotometer (4000−400 cm^{-1}) by using a modern diamond attenuated total reflectance (ATR) accessory method. UV-vis absorption spectra were obtained using a UV-1900i UV-vis spectrophotometer (SHIMADZU). Single-crystal X-ray data collections of the title complexes were completed using a single crystal X-ray diffractometer (Bruker Smart Apex II). The electrical characterization was carried out using a highly sophisticated I-V analyzer (Keithley 4200) instrument in the voltage range of −1 to +1 V. Powder X-ray diffraction (PXRD) data of the complexes were collected by Bruker D8 advance diffractometer with Cu Kα radiation (λ = 1.5418 Å) generated at 40 kV and 40 mA at a scan rate of 0.2 s per step in the range of 2θ from 5° to 40° under ambient condition. Thermogravimetric analysis (TGA) was carried out on a PerkinElmer Pyris Diamond TG/DTA thermal analyzer under a nitrogen atmosphere at the temperature range of 30−800 °C with a heating rate of 10 °C/min.

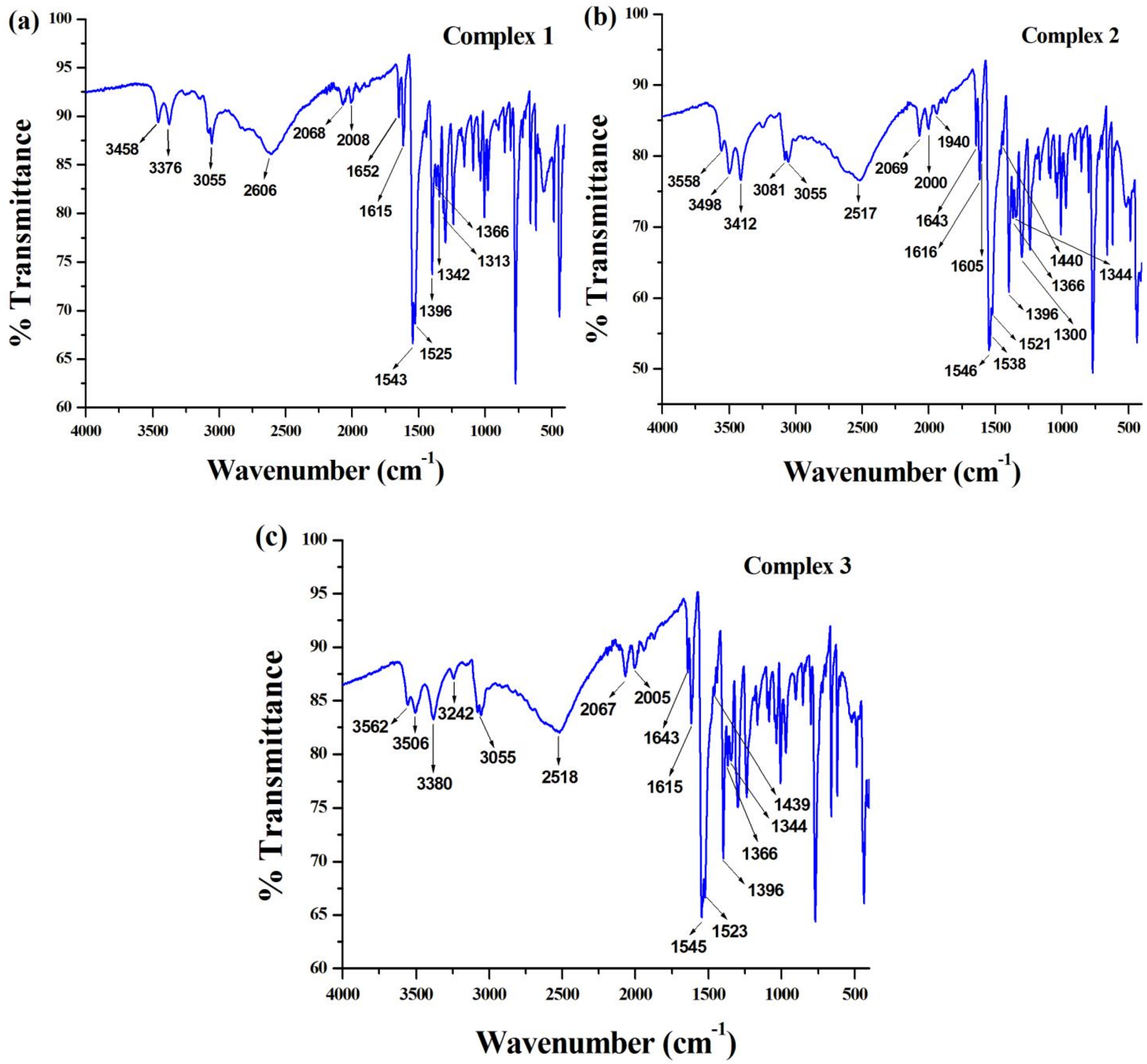

Figure S1. FT-IR Spectra of Complexes **1**, **2** and **3** (a, b and c respectively).

X-ray Crystallographic Analysis

Single crystal X-ray data were collected by using a Bruker SMART APEX II CCD area detector equipped with a graphite monochromated Mo Ka radiation (λ = 0.71073 Å) source in φ and ω scan mode at 295 K for all the complexes. Cell parameter refinement and data reduction for all the complexes were carried out using a Bruker SMART APEX II instrument and Bruker SAINT

Software.[S1] The crystal structures of the title complexes were solved by intrinsic phasing method using SHELXT-2014/5 and refined by full-matrix least squares on F^2 techniques using the SHELXL-2016/6 crystallographic software package.[S2,S3] The CIFs have been deposited with CCDC No. 2223679 (complex **1**), CCDC No. 2223680 (complex **2**) and CCDC No. 2223681 (complex **3**). Selected crystal structure refinement parameters for the complexes are given in Table S1. The important bond lengths and bond angles are presented in Tables S2 and S3, respectively for the complexes.

Table S1. Crystal Data and Structure Refinement Parameters for Complexes 1, 2 and 3

Complex	1	2	3
Empirical formula	$C_{18}H_{17}Cl_5N_6OZn$	$C_{36}H_{36}Cl_{10}N_{12}O_3Cd_2$	$C_{36}H_{36}Cl_{10}N_{12}O_3Hg_2$
Formula Weight	576.02	1264.09	1440.45
Temperature (K)	295	295	295
Wavelength (Å)	0.71073	0.71073	0.71073
Crystal system	Monoclinic	Monoclinic	Monoclinic
space group	$P\ 2_1/n$	$P\ 2_1/n$	$P\ 2_1/n$
a, b, c (Å)	12.2182(10), 14.4158(11), 13.6442(12)	11.2745(8), 14.9594(10), 14.8043(11)	11.2173(6), 14.9175(7), 14.8091(7)
α, β, γ (°)	90, 103.157(3), 90	90, 99.555(2), 90	90, 99.402(2), 90
Volume ($Å^3$)	2340.1(3)	2462.3(3)	2444.8(2)
Z / Density (calc.) (Mg/m^3)	4 / 1.635	2 / 1.705	2 / 1.957
Absorption coefficient (mm^{-1})	1.644	1.455	6.868
F(000)	1160.0	1252.0	1380.0
Crystal size (mm^3)	0.19 × 0.13 × 0.09	0.17 × 0.14 × 0.08	0.19 × 0.13 × 0.09
θ range for data collection	2.021 to 27.188	1.949 to 27.120	1.951 to 27.164
Completeness to θ (%)	100%	100%	100%
Absorption correction	Multi-scan	Multi-scan	Multi-scan
Max. and min. transmission	0.862 and 0.774	0.890 and 0.783	0.539 and 0.359
Refinement method	Full-matrix least-squares on F^2	Full-matrix least-squares on F^2	Full-matrix least-squares on F^2
Data/parameters	5213 / 284	5404 / 295	5404 / 295
Goodness-of-fit on F^2	1.065	1.070	1.047
Final R indices [I >	R_1 = 0.0295,	R_1 = 0.0263,	R_1 = 0.0606,

2σ(I)]	wR_2 = 0.0710	wR_2 = 0.0666	wR_2 = 0.1528
R indices (all data)	R_1 = 0.0349, wR_2 = 0.0742	R_1 = 0.0308, wR_2 = 0.0691	R_1 = 0.0733, wR_2 = 0.1621
Largest diff. peak and hole (e.Å^{-3})	0.665 and -0.415	0.518 and -0.413	1.218 and -1.991

$R_1=\sum||F_o|-|F_c||/\sum|F_o|$, $wR_2 = [\sum\{(F_o^2-F_c^2)^2\}/\sum\{w(F_o^2)^2\}]^{1/2}$ $w = 1/\{\sigma^2(F_o^2) + (aP)^2 + bP\}$; where, $P = (F_o^2 + 2F_c^2)/3$.

For Complex **1:** a = 0.0303 and b = 1.4103. For Complex **2:** a = 0.0307 and b = 1.4633. For Complex **3:** a = 0.0886 and b = 13.3175.

Table S2. Selected Bond Distances [Å] for Complexes 1, 2 and 3

	Bonds	Distance (Å)	Bonds	Distance (Å)
Complex **1**	Zn1─Cl4	2.2897(7)	Zn1─Cl2	2.2440(7)
	Zn1─Cl1	2.2658(7)	Zn1─Cl3	2.2601(6)
Complex **2**	Cd1─Cl4	2.4397(7)	Cd1─Cl2	2.4519(7)
	Cd1─Cl1	2.4461(8)	Cd1─Cl3	2.4790(7)
Complex **3**	Hg1─Cl4	2.458(2)	Hg1─Cl2	2.462(2)
	Hg1─Cl1	2.540(2)	Hg1─Cl3	2.451(2)

Table S3. Selected Bond Angles [°] for Complexes 1, 2 and 3

	Bond angles	Value (°)	Bond angles	Value (°)
Complex **1**	Cl2─Zn1─Cl4	109.09(3)	Cl1─Zn1─Cl2	118.90(3)
	Cl3─Zn1─Cl4	111.87(2)	Cl1─Zn1─Cl3	106.61(3)
	Cl1─Zn1─Cl4	103.44(2)	Cl2─Zn1─Cl3	106.99(3)
Complex **2**	Cl2─Cd1─Cl4	114.23(3)	Cl1─Cd1─Cl2	112.19(3)
	Cl3─Cd1─Cl4	106.84(2)	Cl1─Cd1─Cl3	104.40(3)
	Cl1─Cd1─Cl4	112.44(3)	Cl2─Cd1─Cl3	105.86(3)
Complex **3**	Cl2─Hg1─Cl4	113.87(8)	Cl1─Hg1─Cl2	103.72(8)
	Cl3─Hg1─Cl4	114.98(9)	Cl1─Hg1─Cl3	104.58(7)
	Cl1─Hg1─Cl4	104.32(8)	Cl2─Hg1─Cl3	113.69(9)

Table S4. Geometrical Parameters for the Hydrogen Bonds of Complexes 1, 2 and 3

D─H···A	D─H [Å]	H···A [Å]	D···A [Å]	D─H···A [°]	Symmetry
Complex **1**					
O1─H1C···Cl1	0.8500	2.3300	3.1594(19)	166.00	1/2-x, 1/2+y,1/2-z
O1─H1B···Cl4	0.8500	2.2600	3.110(2)	179.00	-
N1─H1···Cl5	0.8600	2.2500	3.0624(19)	157.00	1+x,y,z
N1─H1···N2	0.8600	2.3200	2.682(2)	106.00	-
N5─H5···O1	0.8600	1.9400	2.711(2)	148.00	-
N5─H5···N4	0.8600	2.3400	2.704(2)	106.00	-
N6─H6···Cl5	0.8600	2.2700	3.0650(2)	155.00	1+x,y,z
N6─H6···N2	0.8600	2.3000	2.671(2)	106.00	-
C2─H2···Cl3	0.9300	2.6400	3.554(3)	168.00	1-x,1-y,1-z
C17─H17···Cl2	0.9300	2.6000	3.439(3)	150.00	3/2+x,3/2-y,1/2+z
C16─H16···Cl5	0.9300	2.8200	3.637(3)	147.00	3/2-x,1/2+y,3/2-z
C13─H13···Cl3	0.9300	2.6500	3.500(2)	153.00	1-x,2-y,1-z
Complex **2**					
O1─H1C···Cl3	0.8500	2.2800	3.110(2)	165.00	-
O1─H1B···Cl4	0.8500	2.4100	3.224(2)	160.00	1/2-x,1/2+y,1/2-z
N1─H1···Cl5	0.8600	2.2300	3.044(2)	157.00	1+x,y,z
N1─H1···N2	0.8600	2.3200	2.689(2)	106.00	-
O2─H2A···Cl1	0.8500	2.2900	3.136(5)	171.00	1/2-x,1/2+y,1/2-z
O2─H2B···Cl2	0.8500	2.4600	3.305(5)	170.00	1-x,1-y,1-z
N5─H5···O1	0.8600	1.9900	2.766(3)	150.00	-
N5─H5···N4	0.8600	2.3200	2.692(3)	106.00	-
N6─H6···Cl5	0.8600	2.2400	3.050(2)	156.00	1+x,y,z
N6─H6C···N2	0.8600	2.2900	2.665(3)	107.00	-
C17─H17···Cl2	0.9300	2.7300	3.621(3)	161.00	3/2+x,1/2-y,1/2+z
C16─H16···Cl5	0.9300	2.7100	3.534(3)	147.00	3/2-x,-1/2+y,3/2-z
C10─H10···Cl3	0.9300	2.8200	3.731(2)	166.00	-
Complex **3**					
N1─H1···Cl5	0.8600	2.2300	3.044(7)	158.00	1+x,y,z
N1─H1···N2	0.8600	2.3300	2.693(8)	106.00	-
O1─H1B···Cl1	0.8500	2.2500	3.098(9)	175.00	-
O1─H1C···Cl3	0.8500	2.4100	3.216(8)	158.00	1/2-x,1/2+y,1/2-z
O2─H2A···Cl2	0.8500	2.3200	3.09(2)	152.00	1/2-x,-1/2+y,1/2-z
O2─H2B···Cl4	0.8500	2.4200	3.240(19)	162.00	1-x,1-y,1-z
N5─H5···O1	0.8600	1.9700	2.754(10)	150.00	-
N5─H5···N4	0.8600	2.3300	2.695(10)	106.00	-
N6─H6···Cl5	0.8600	2.2400	3.051(8)	156.00	1+x,y,z
N6─H6···N2	0.8600	2.2900	2.662(10)	106.00	-
C17─H17···Cl4	0.9300	2.7300	3.628(9)	161.00	3/2+x,3/2-y,1/2+z
C16─H16···Cl5	0.9300	2.6700	3.499(10)	149.00	3/2-x,-1/2+y,3/2-z
C10─H10···Cl1	0.9300	2.8000	3.711(9)	166.00	-

Table S5. Geometrical Parameters (Å, °) for the Anion···π Interactions for the Title Complexes

Y─X(I)···Cg(J)	X···Cg [Å]	Y···Cg [Å]	Y─X···Cg (°)	Symmetry
Complex **1**				
Zn(1)─Cl(1)[2]···Cg(1)	3.5829(12)	5.3919(11)	133.11(3)	-1/2+x,3/2-y,-1/2+z
Zn(1)─Cl(2)[2]···Cg(2)	3.5103(11)	5.3535(9)	135.84(3)	-1+x,y,z
Zn(1)─Cl(3)[2]···Cg(4)	3.4546(11)	5.0581(10)	123.16(3)	-1+x,y,z
Zn(1)─Cl(4)[2]···Cg(2)	3.3511(10)	5.1296(9)	129.88(3)	-1/2+x,3/2-y,-1/2+z
Complex **2**				
Cd(1)─Cl(1)[2]···Cg(3)	3.7185(12)	5.4511(11)	122.99(3)	-1/2+x,1/2-y,-1/2+z
Cd(1)─Cl(2)[2]···Cg(2)	3.2754(10)	5.6675(9)	163.25(3)	-1+x,y,z
Cd(1)─Cl(3)[2]···Cg(2)	3.3779(11)	4.4908(9)	98.97(3)	-1/2+x,1/2-y,-1/2+z
Cd(1)─Cl(4)[2]···Cg(1)	3.6781(14)	5.5516(12)	129.18(3)	-1/2+x,1/2-y,-1/2+z
Complex **3**				
Hg(1)─Cl(1)[2]···Cg(2)	3.366(4)	4.508(3)	98.54(8)	-1/2+x,3/2-y,-1/2+z
Hg(1)─Cl(2)[2]···Cg(3)	3.691(4)	5.453(3)	123.58(9)	-1/2+x,3/2-y,-1/2+z
Hg(1)─Cl(3)[2]···Cg(1)	3.660(5)	5.578(4)	130.74(11)	-1/2+x,3/2-y,-1/2+z
Hg(1)─Cl(4)[2]···Cg(2)	3.251(4)	5.637(3)	161.61(11)	-1+x,y,z

Cg(j) denotes centroid of jth ring of the title complexes. For Complex **1**: Cg(1) is the centroid of [N1/C1/C2/C3/C4/C5] ring; Cg(2) is the centroid of [N2/C6/N4/C8/N3/C7] ring; and Cg(4) is the centroid of [N6/C17/C16/C15/C14/C18] ring. For Complex **2**: Cg(1) is the centroid of [N1/C1/C2/C3/C4/C5] ring; Cg(2) is the centroid of [N2/C6/N4/C8/N3/C7] ring; and Cg(3) is the centroid of [N5/C9/C13/C12/C11/C10] ring. For Complex **3**: Cg(1) is the centroid of [N1/C1/C2/C3/C4/C5] ring; Cg(2) is the centroid of [N2/C6/N4/C8/N3/C7] ring and Cg(3) is the centroid of [N5/C9/C13/C12/C11/C10] ring.

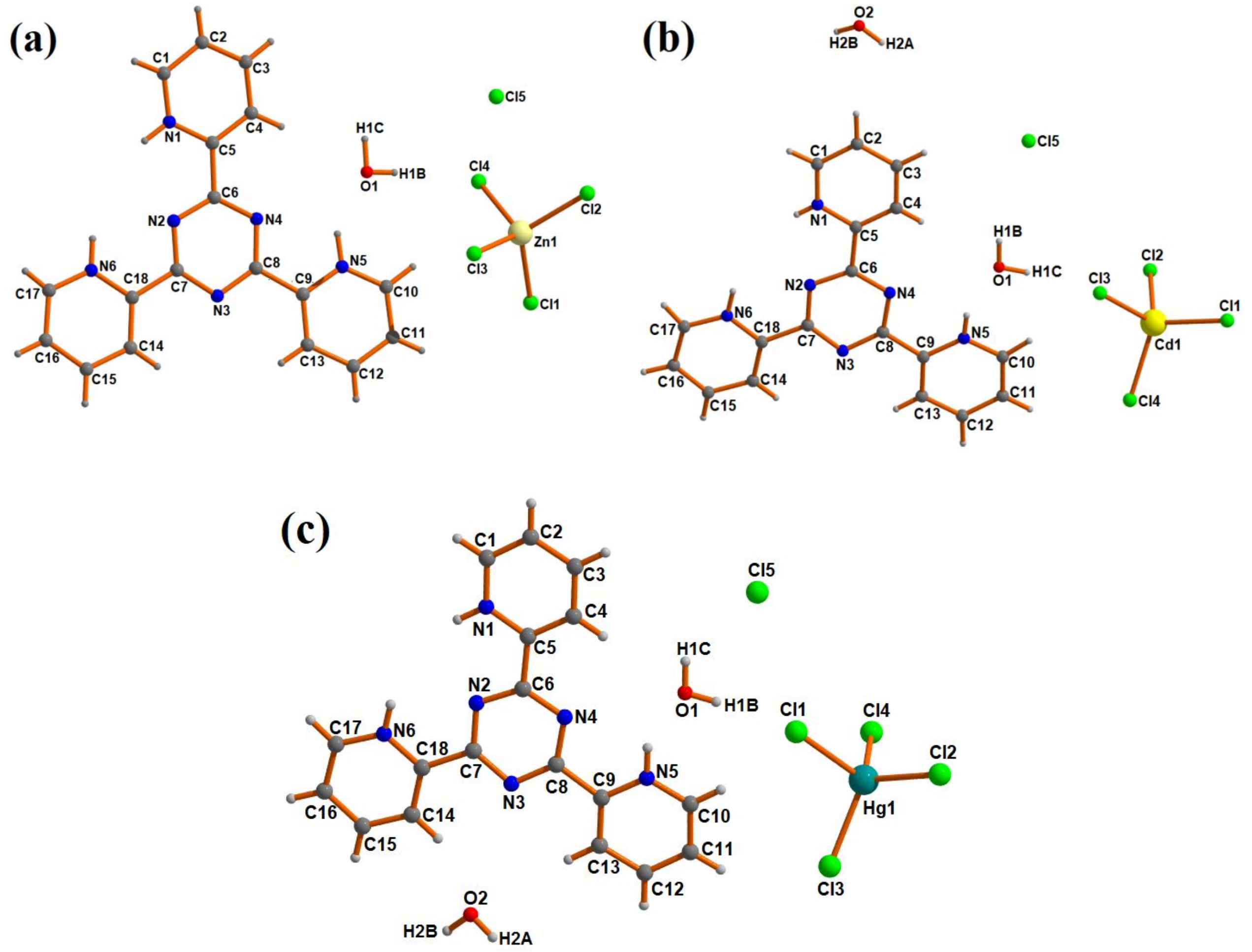

Figure S2. Asymmetric Units of Complexes **1**, **2** and **3** (a, b and c respectively).

Powder X-Ray Diffraction Analysis

PXRD has been carried out at room temperature (295 K) with the powdered sample of the complexes (**1**, **2** and **3**). The bulk purity of the complexes has been confirmed by the PXRD pattern analysis. The major peaks of the PXRD pattern of the synthesized complexes match well with the simulated pattern obtained from the single crystal data, representing phase purity of the bulk as shown in Figure S3. The minor shifts and the differences in the intensities might be attributed due to the baseline drift of the PXRD diffractometer and the changes either in crystal lattice orientation with respect to an inertial frame of reference or sample thickness.[S4,S5]

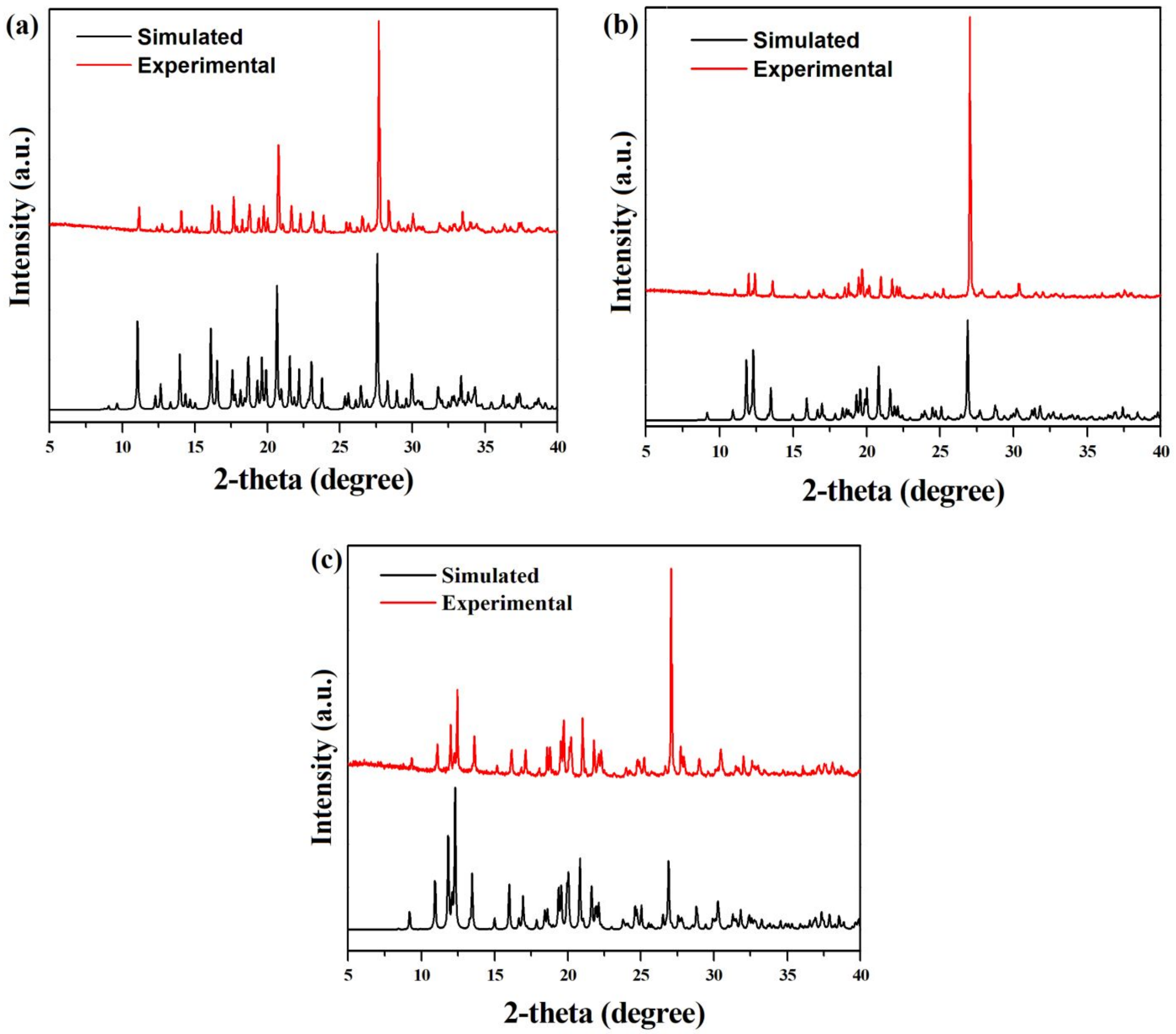

Figure S3. PXRD Patterns of Experimental Powder XRD (red) and Simulated Pattern from Single Crystal Data (black) for Complexes **1**, **2** and **3** (a, b and c respectively).

Thermogravimetric Analysis (TGA) of the Title Complexes

Thermo gravimetric (TG) analysis was carried out for all three complexes (**1-3**) to acquire a structural insight into the number of water molecules present in the complexes. The TG curves for all three complexes (**1-3**) are shown in Figure S3 (a-c). For complex **1**, it clearly shows that the complex is thermally stable up to 136 ºC. In the temperature range from 136 to 168 ºC, it loses one non-coordinated water molecule (calculated mass loss 3.12%, found 3.14%). For

complex **2**, it clearly exhibits that the complex is thermally stable up to 124 ºC. For complex **2**, in the temperature range 124 to 179 ºC respectively that clearly indicative for the presence of 1.5 water molecules per asymmetric unit (calculated mass loss 4.27%, found 4.28%). Complex **3** exhibits thermal stability up to 73 ºC and then in the thermal range 63 to 146 ºC it shows a declination (calculated mass loss 3.75%, found 3.74%) which is to be found the presence of 1.5 water molecules per asymmetric unit as that of complex **2**. Actually, three water molecules are utilized by two asymmetric units of complexes **2** and **3**.

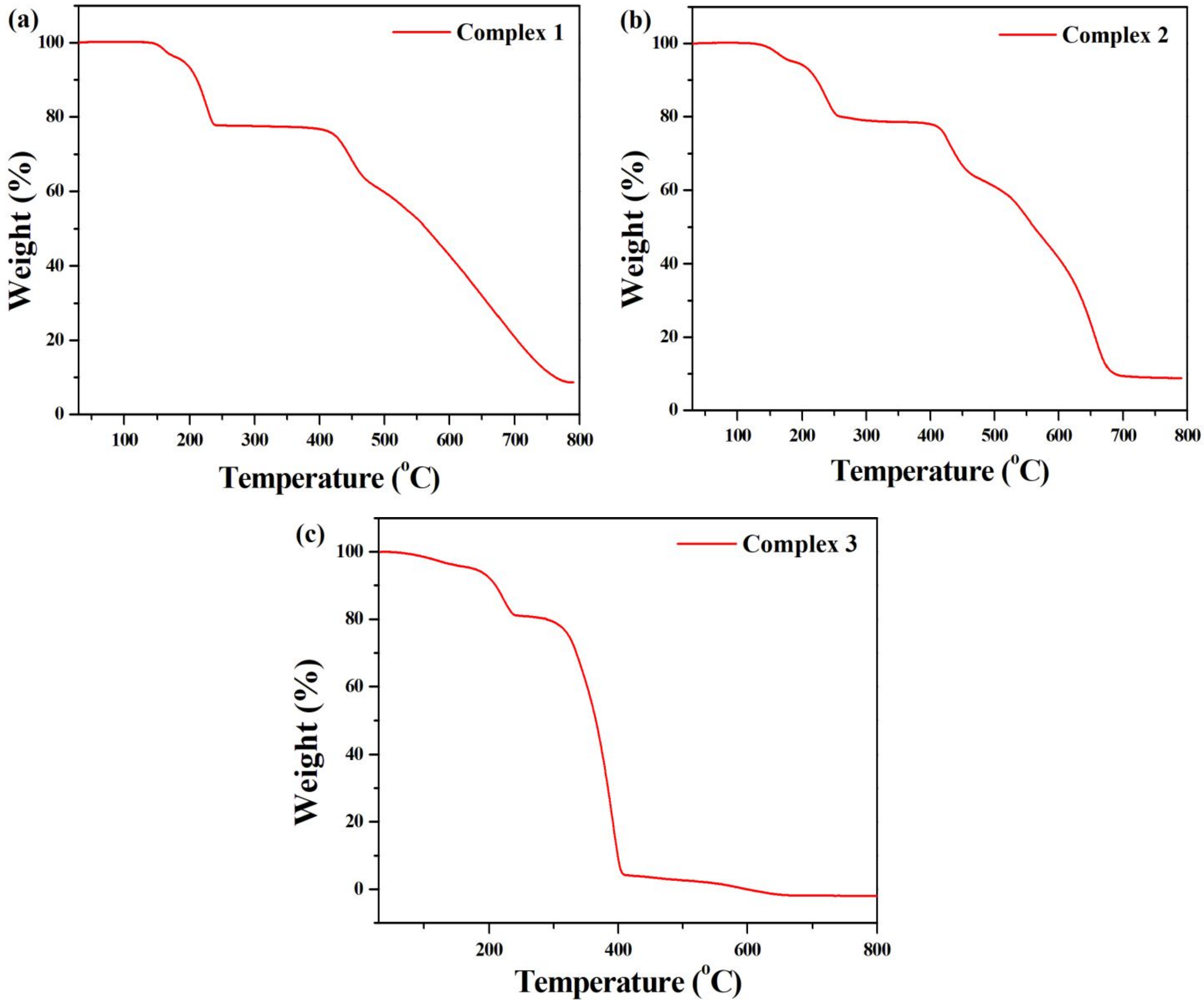

Figure S4. Thermal analysis of complexes **1**, **2** and **3** (a-c).

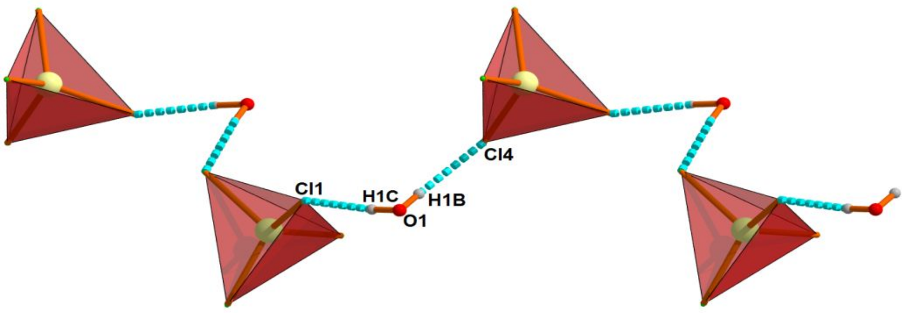

Figure S5. Formation of a 1D water-anion cluster in complex **1**.

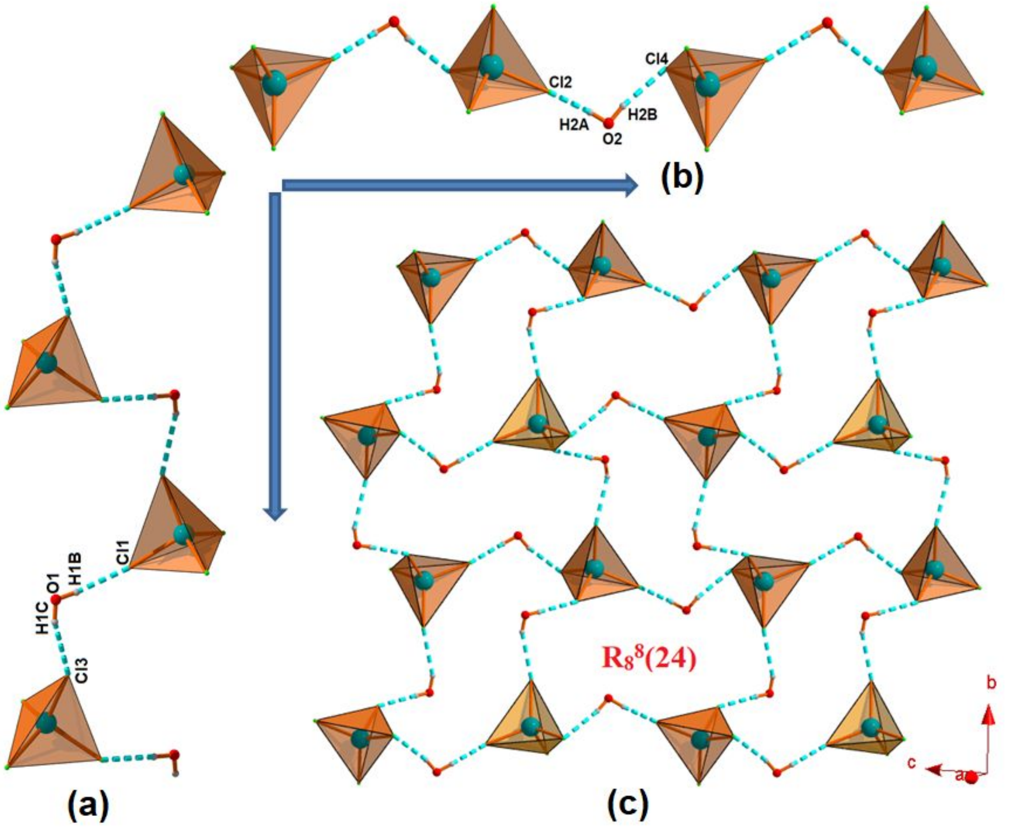

Figure S6. (a) Formation of a 1D water-anion cluster along (010) direction. (b) Propagation of another 1D water-anion cluster along (001) direction. (c) Extension of water-anion cluster in *bc*-plane in complex **3**.

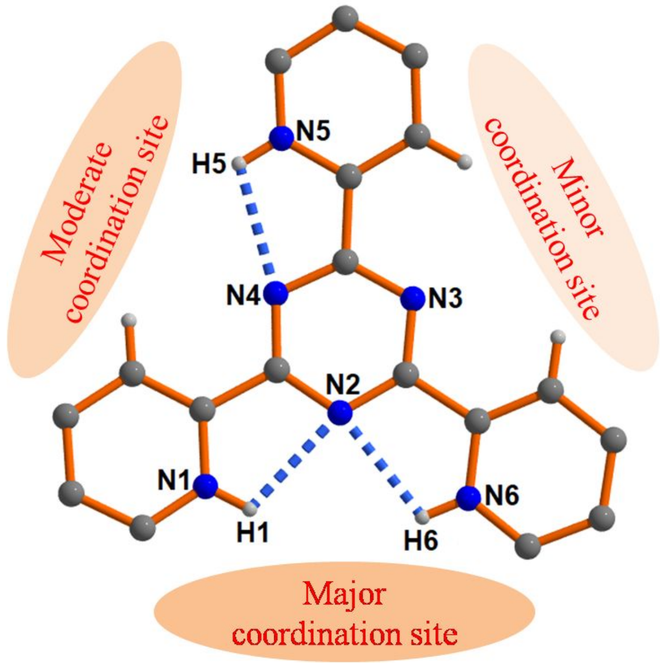

Figure S7. Intramolecular hydrogen bonding interactions restrict the neutral coordination sites of the tptz ligand.

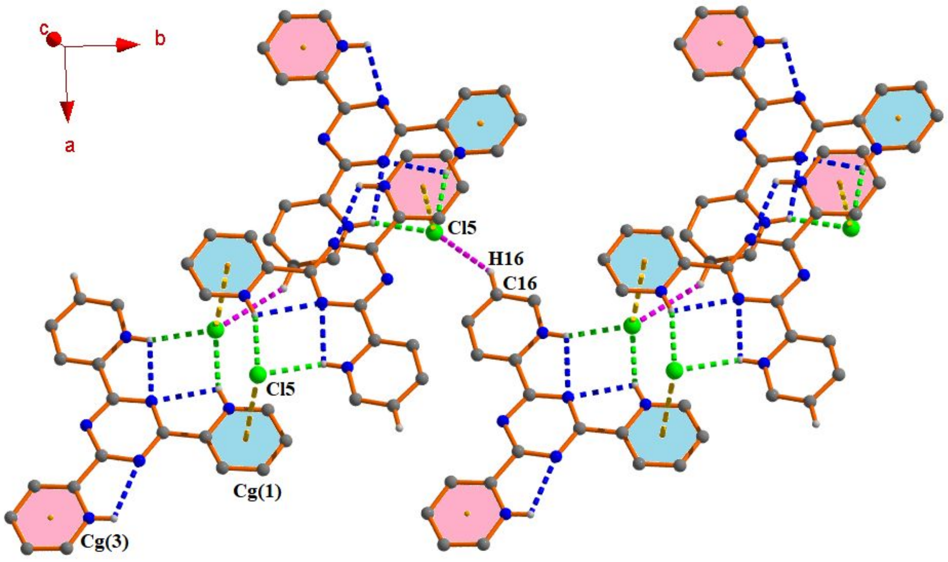

Figure S8. View of 2D layered assembly through anion···π$^+$ and hydrogen bonding interactions in complex **1**.

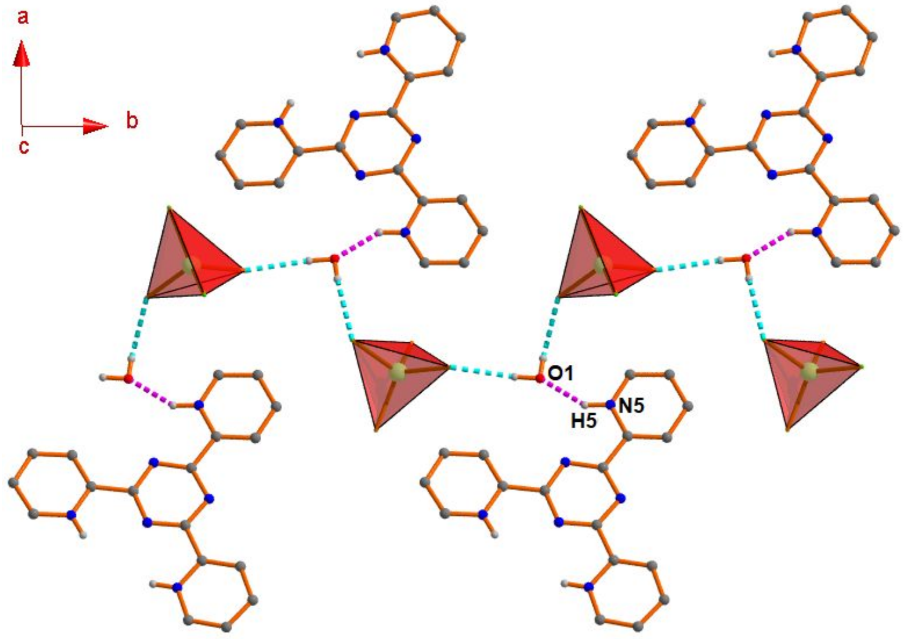

Figure S9. View of 1D tape through hydrogen bonding interactions in complex **1**.

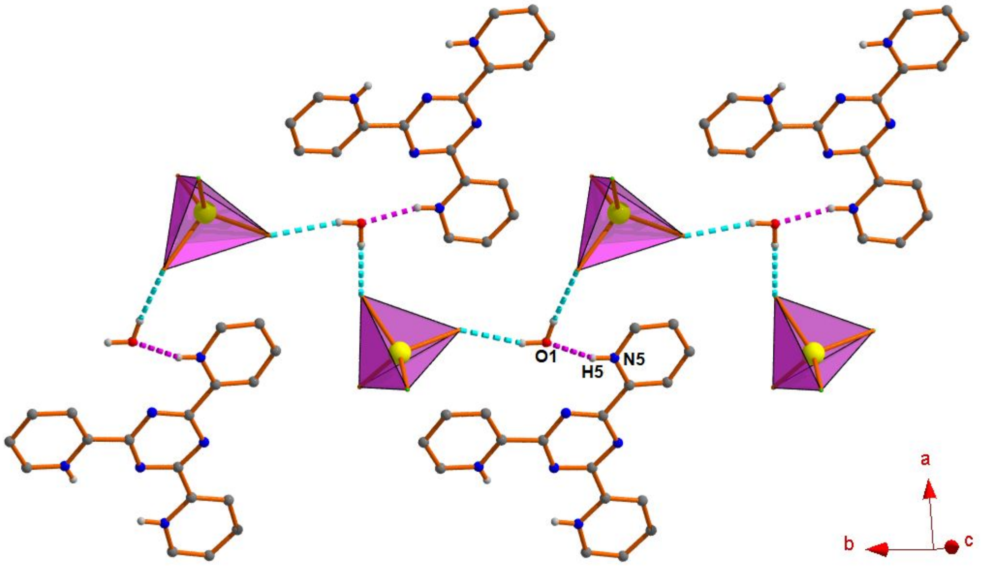

Figure S10. Perspective view of 1D tape through hydrogen bonding interactions in complex **2**.

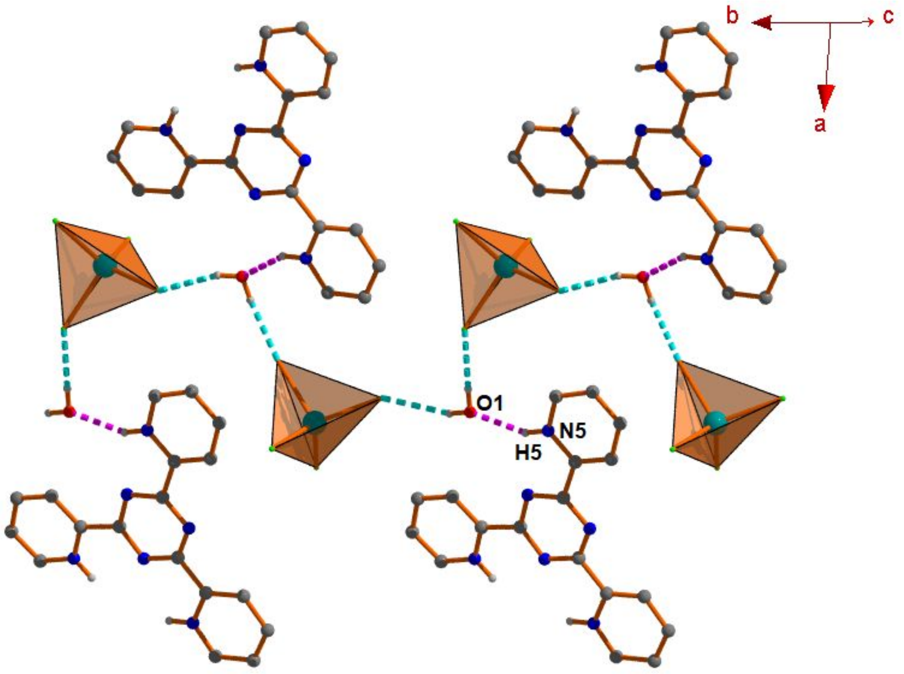

Figure S11. Perspective view of 1D tape through hydrogen bonding interactions in complex **3**.

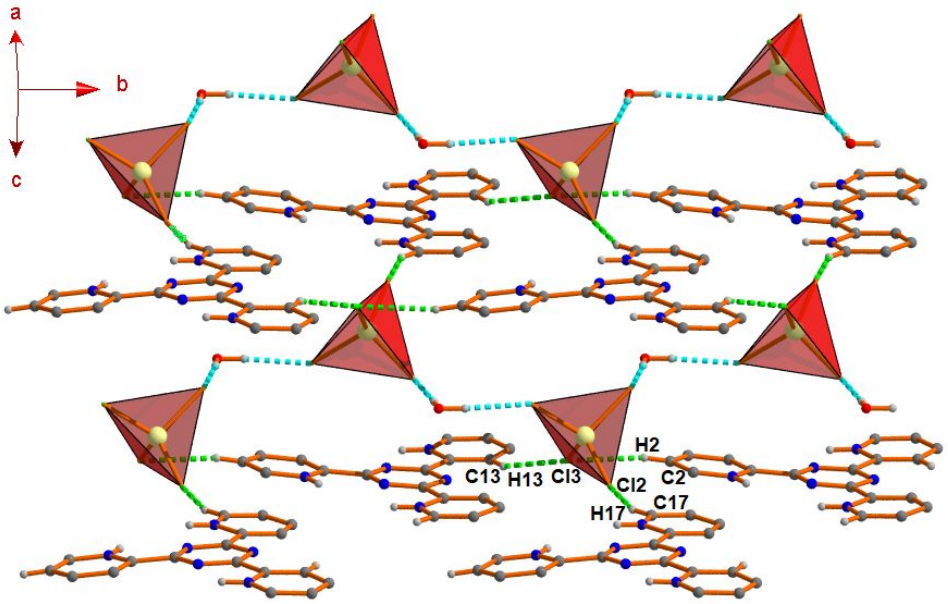

Figure S12. Formation of 2D layer incorporating water-anion cluster and C–H···Cl interactions in complex **1**.

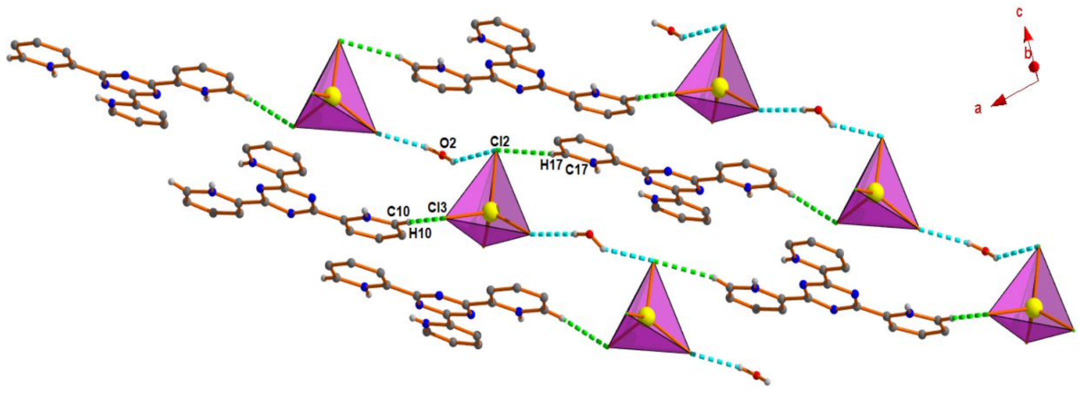

Figure S13. 2D layer incorporating water-anion cluster and C–H···Cl interactions in **2**.

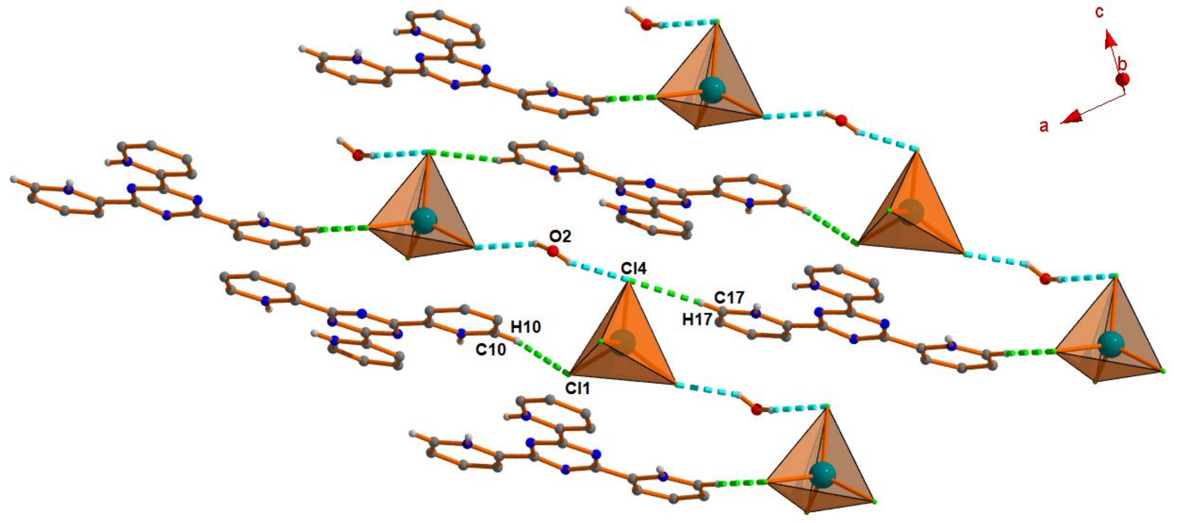

Figure S14. 2D layer incorporating water-cluster and C–H···Cl interactions in **3**.

Hirshfeld Surface Analysis

Hirshfeld surfaces[S6–S8] and the related two-dimensional (2D) fingerprint[S9–S11] plots presented in this paper were generated using Crystal Explorer 21.5,[S12] with bond lengths to hydrogen atoms set to standard values. For each point on the Hirshfeld surface, two distances, d_e (the distance

from the point to the nearest nucleus external to the surface) and d_i (the distance to the nearest nucleus internal to the surface), are defined. The normalized contact distance (d_{norm}) based on d_e, d_i and the van der Waal radii of the atom is given by the following equation.

$$d_{norm} = \frac{(d_i - r_i^{vdw})}{r_i^{vdw}} + \frac{(d_e - r_e^{vdw})}{r_e^{vdw}} \quad \text{(S1)}$$

The value of the d_{norm} is negative or positive when intermolecular contacts are shorter or longer than the van der Waals separations, respectively. The parameter d_{norm} displays a surface with a red-white-blue colour scheme, where red highlights shorter contacts, white is used for contacts around the van der Waals separation and blue regions are devoid of close contacts. For a given crystal structure and set of spherical atomic electron densities, the Hirshfeld surface is said to be unique.[S13]

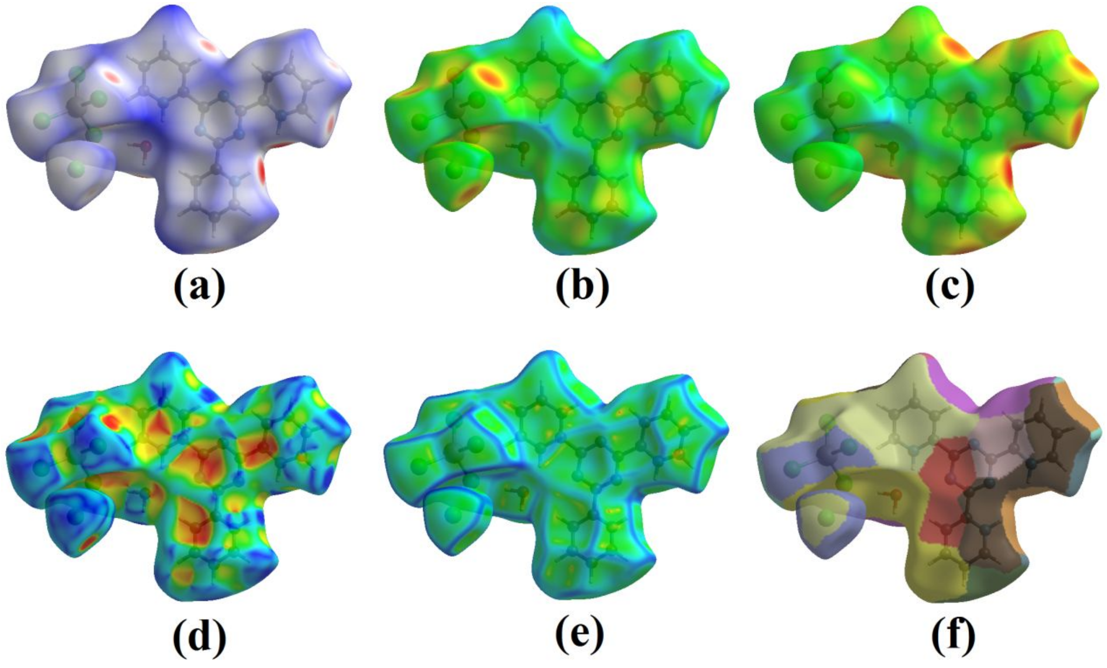

Figure S15. Hirshfeld surfaces mapped with (a) d_{norm} (b) d_e (c) d_i (d) shape-index (e) curvedness and (f) fragment patches for the complex **1**.

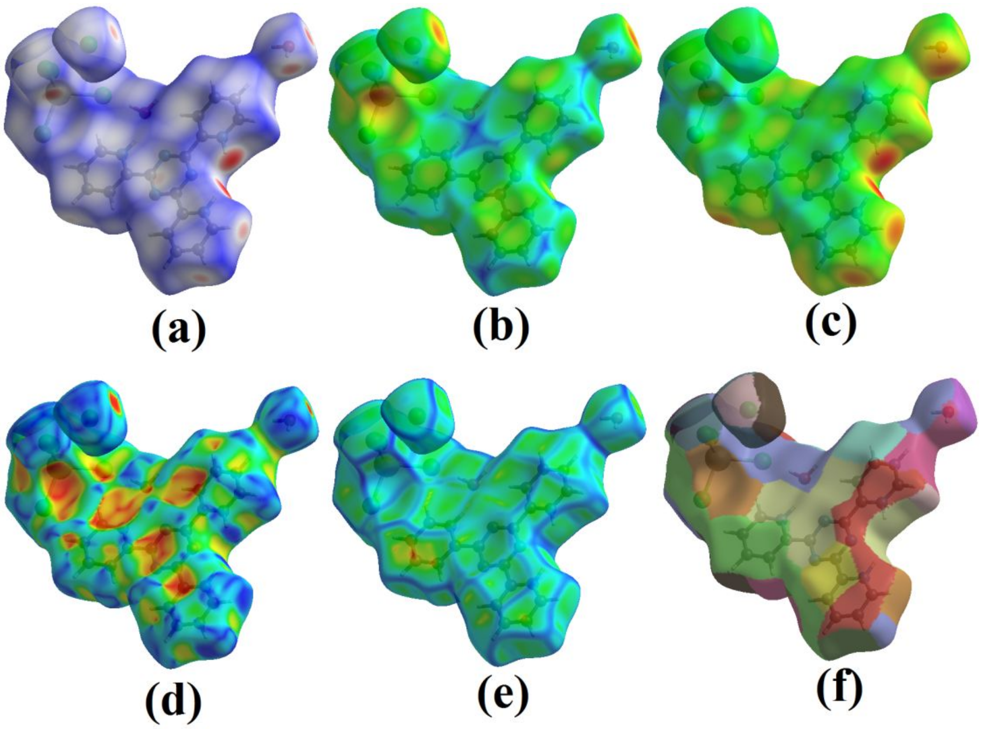

Figure S16. Hirshfeld surfaces mapped with (a) d_{norm} (b) d_e (c) d_i (d) shape-index (e) curvedness and (f) fragment patches for the complex **2**.

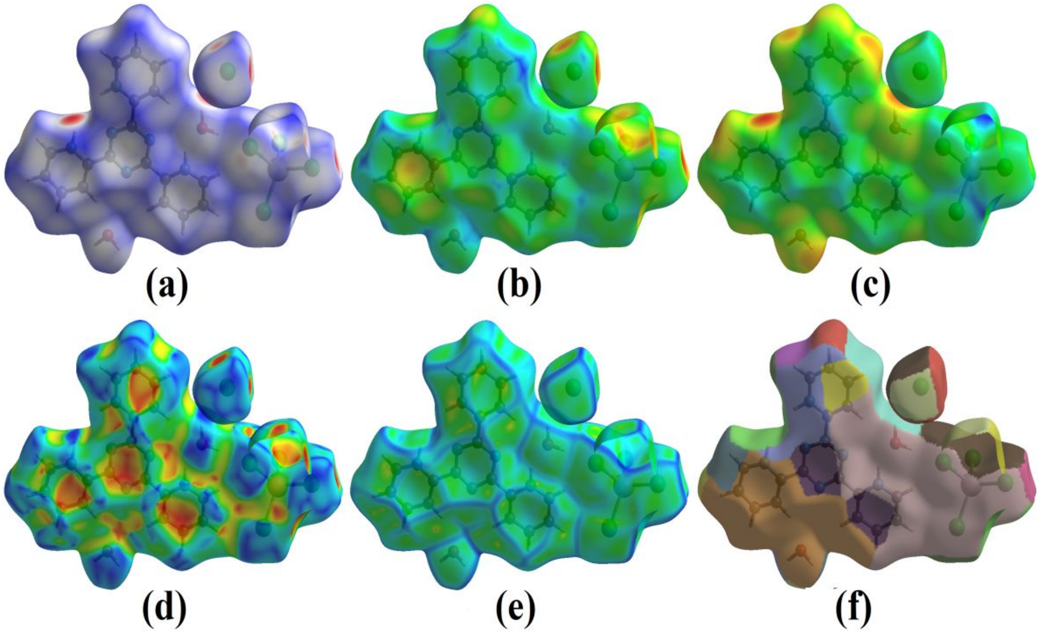

Figure S17. Hirshfeld surfaces mapped with (a) d_{norm} (b) d_e (c) d_i (d) shape-index (e) curvedness and (f) fragment patches for the complex **3**.

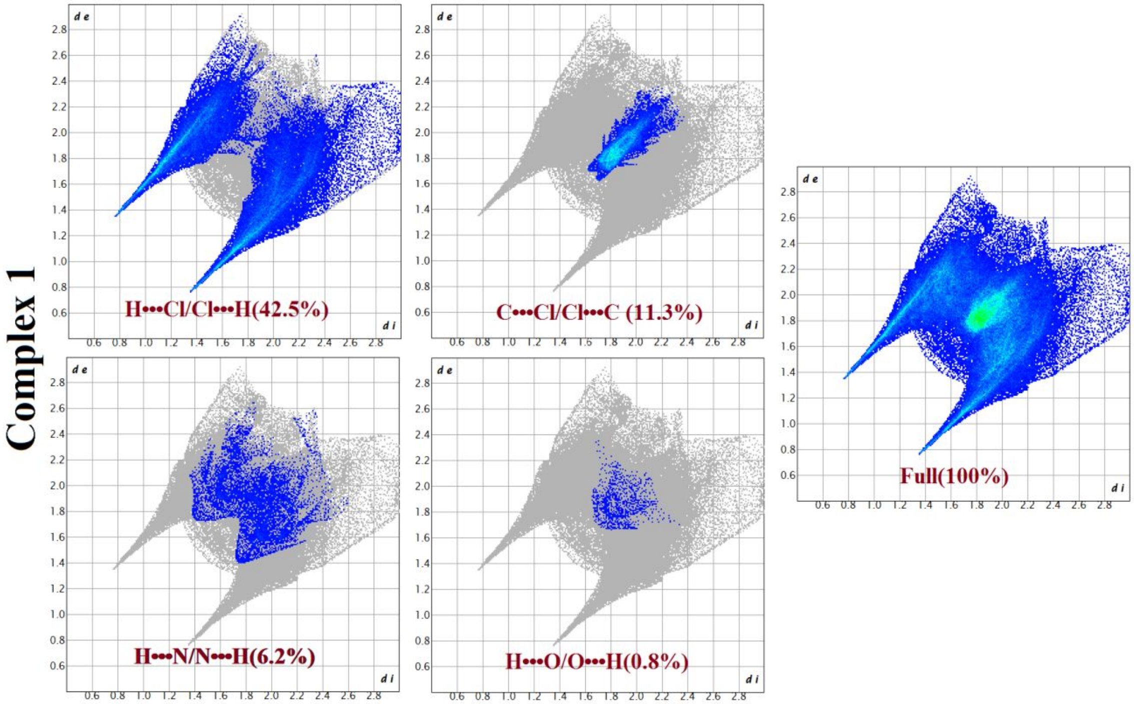

Figure S18. Fingerprint plots (Full) and decomposed plots for each individual interaction in complex **1**.

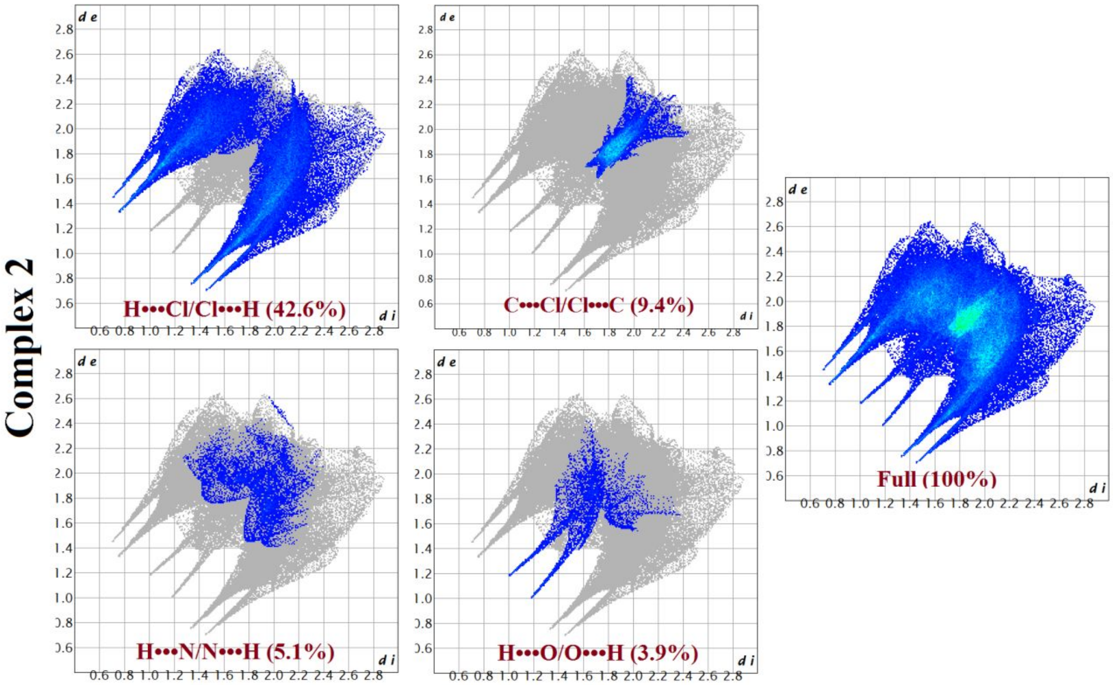

Figure S19. Fingerprint plots (Full) and decomposed plots for each individual interaction in complex **2**.

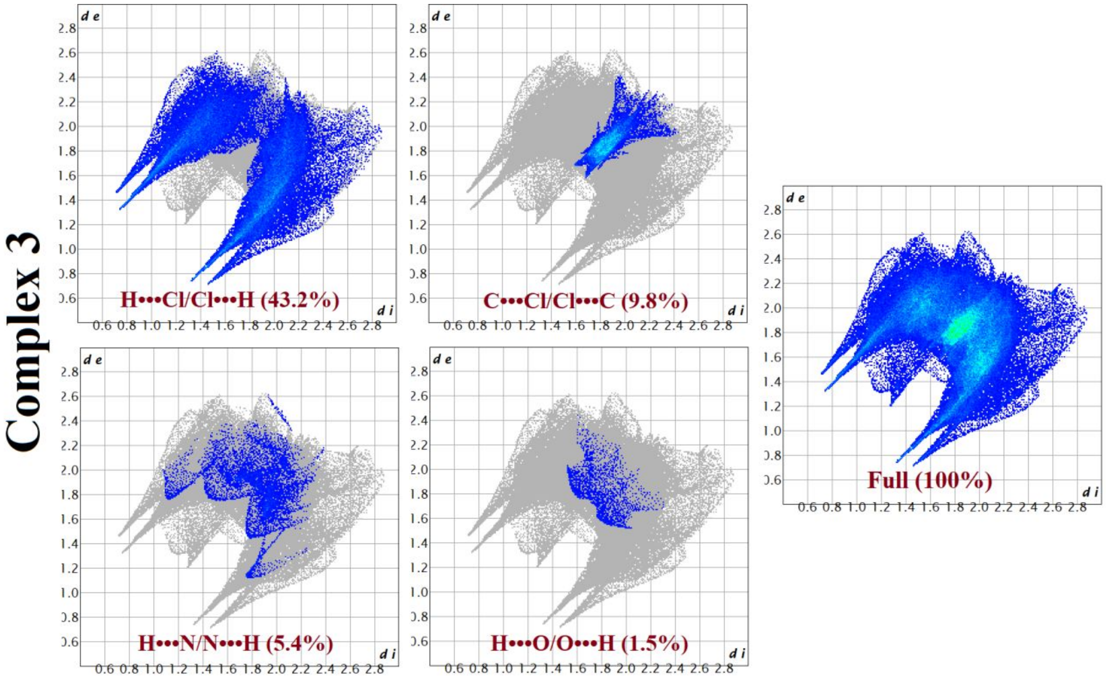

Figure S20. Fingerprint plots (Full) and decomposed plots for each individual interaction in complex **3**.

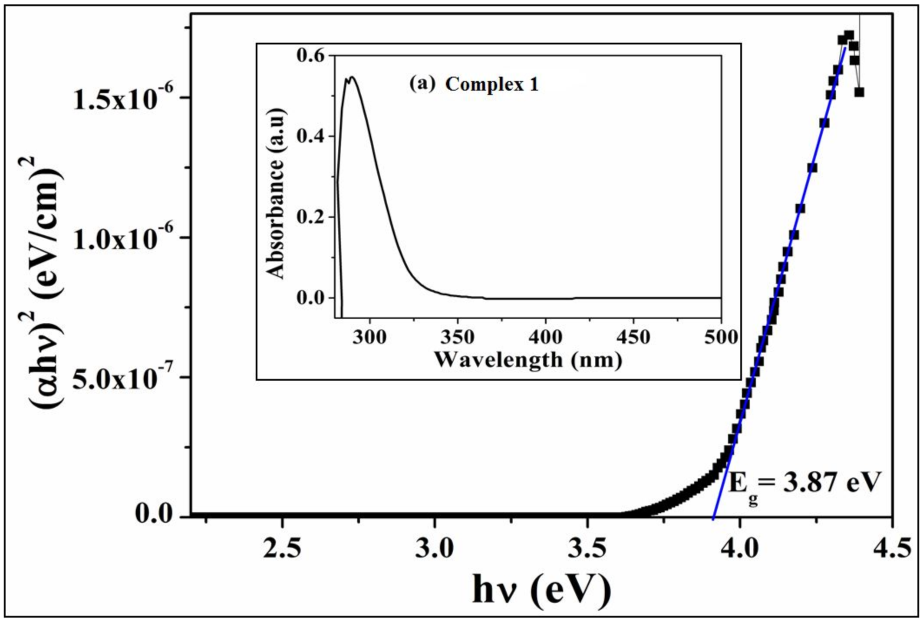

Figure S21. UV-Vis spectrum of complex **1** (inset) and Tauc's plot.

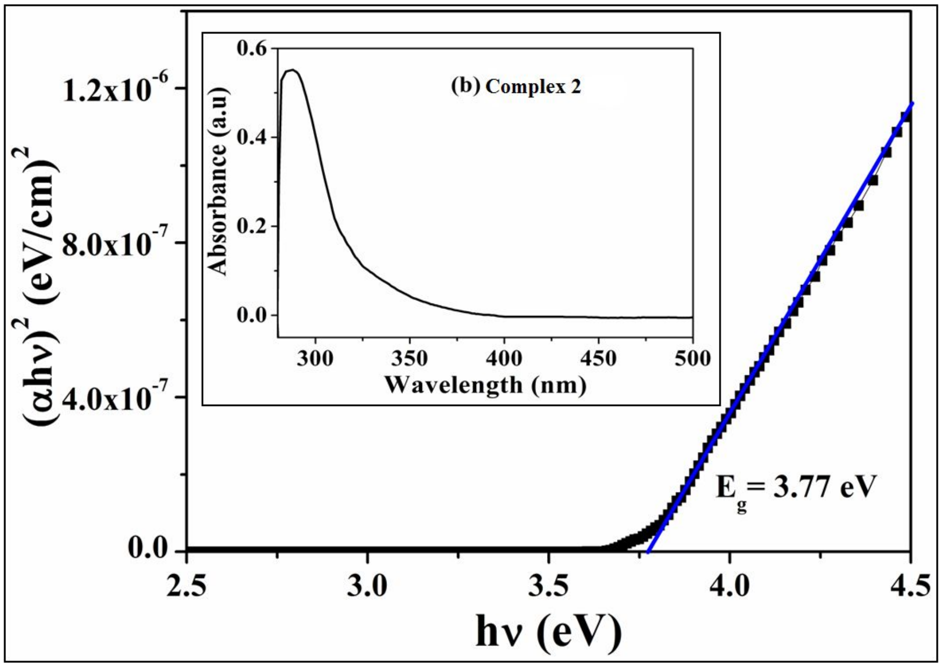

Figure S22. UV-Vis spectrum of complex **2** (inset) and Tauc's plot.

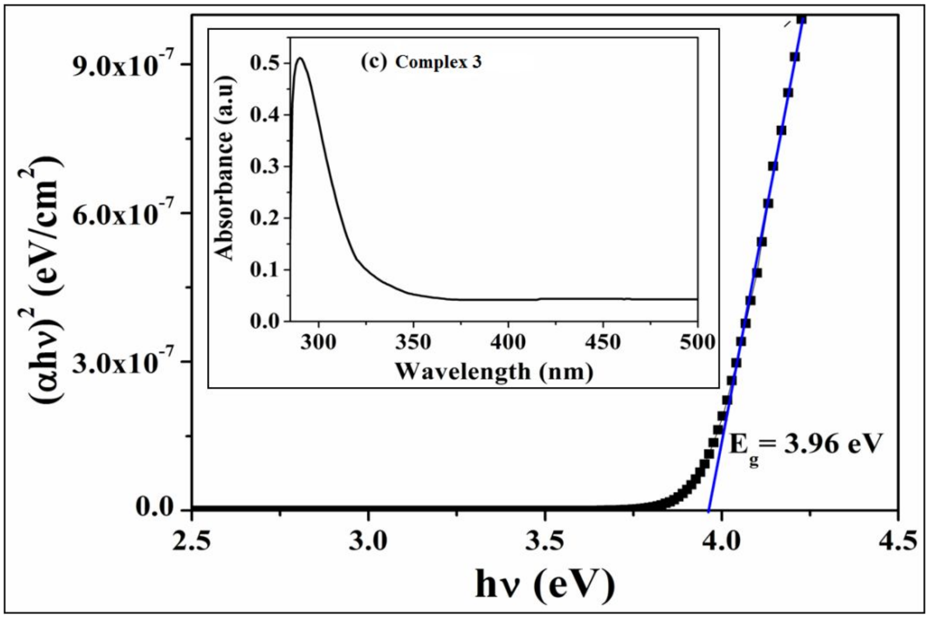

Figure S23. UV-Vis spectrum of complex **3** (inset) and Tauc's plot.

Thin Film Fabrication from the Precursor Complexes

Recent development in SBD fabrication shows that functional materials are must be thin film form for simple use and ease of device parameters. Thin film technology bridges in between macro and molecular systems where dimension of the material is decreased widely for accurate device fabrication. With this overview thin film preparation from a particular complex has been started. Usually, spin-coating method is ideal to make a film for low cost, minimum material, substrate flexibility, and tunable thickness. In the present work, three precursor complexes (**1-3**) were separately deposited onto properly clean ITO substrates (resistance ~10-12 Ω/sq cm, surface area 1.0×1.0 cm^2) by spin-coating method. About 3 to 4 mg of each complex were dispersed in DMF (ratio 1:4) and ultrasonicated for 45 minutes. Few drops of this precursor solution were dropped onto the center of ITO and spinned at 700 rpm and then 1000 rpm for 5 and 7 minutes respectively. The resulting thin films were dried in inert atmosphere and reserved in desiccators.

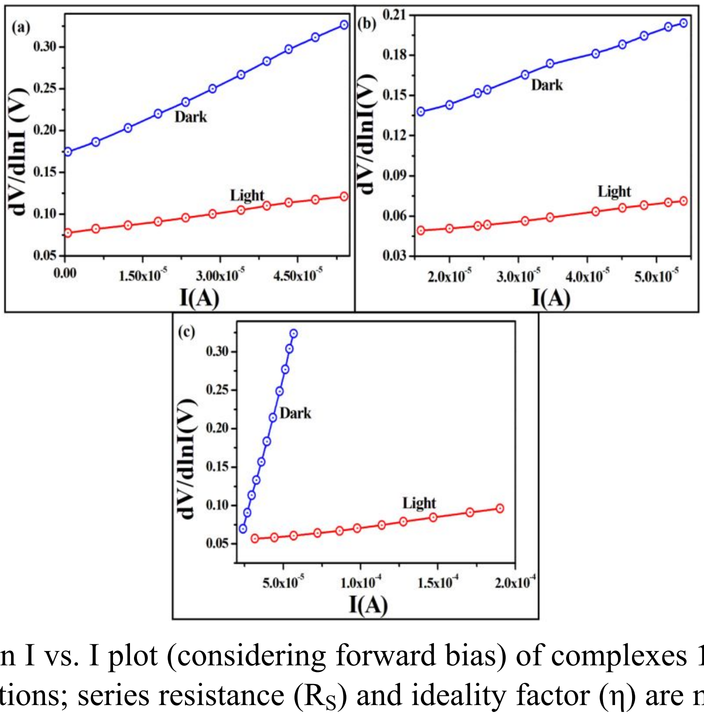

Figure S24. dV/dln I vs. I plot (considering forward bias) of complexes **1-3** (a-c) under dark and illumination conditions; series resistance (R_S) and ideality factor (η) are measured from the slope and intercept of the plot.

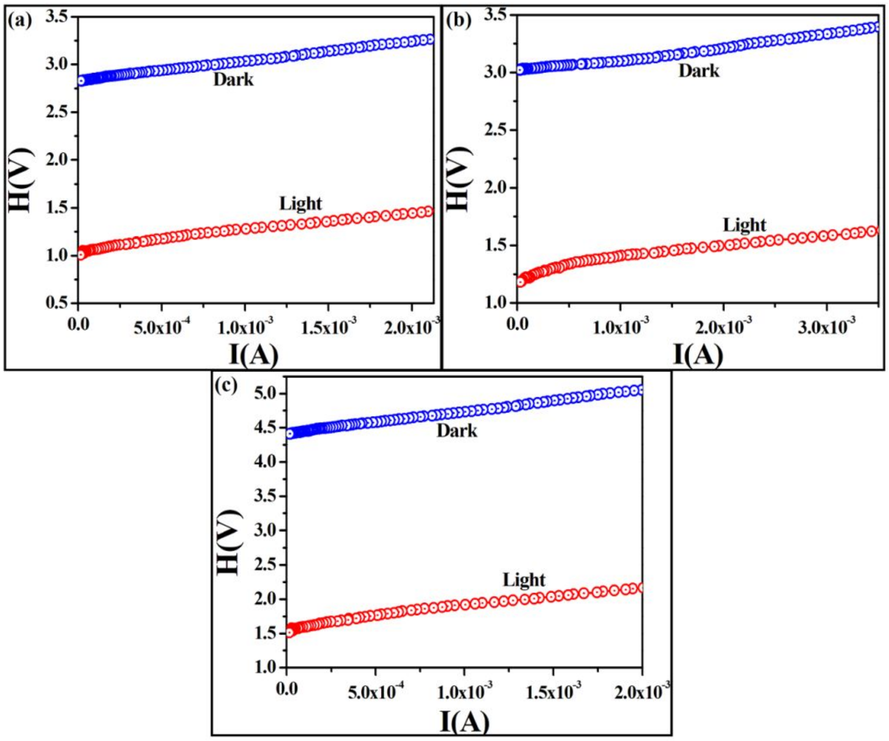

Figure S25. H (I) vs. I plot of complexes **1-3** (a-c) under dark and illumination condition. Here, H(I) represents difference in Ohmic and Schottky contact of the device.

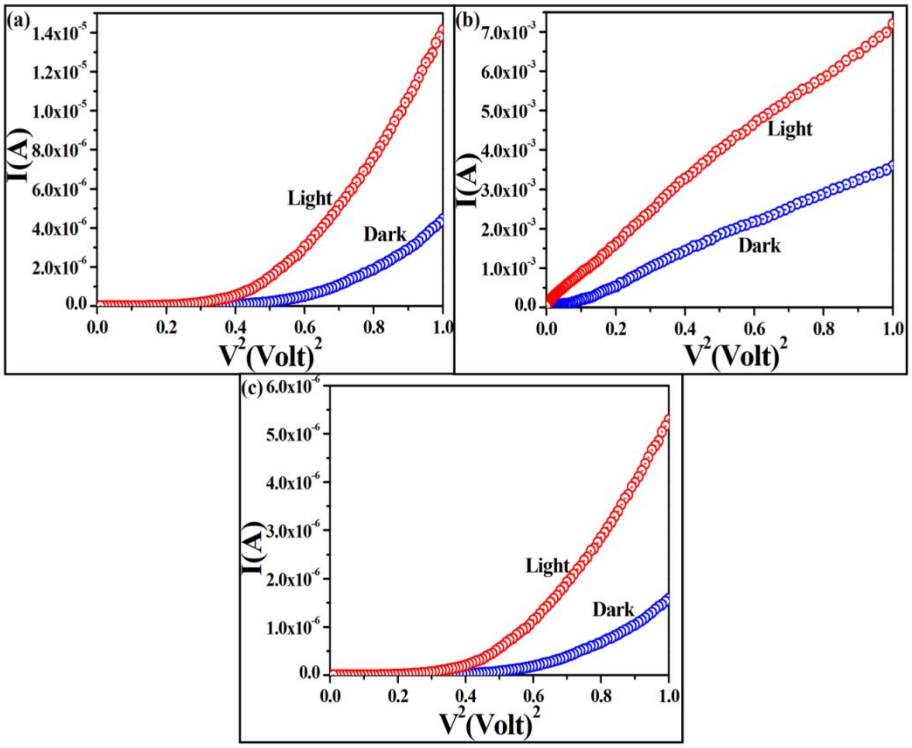

Figure S26. I vs V^2 plot of complexes **1-3** (a-c) under dark and illumination condition.

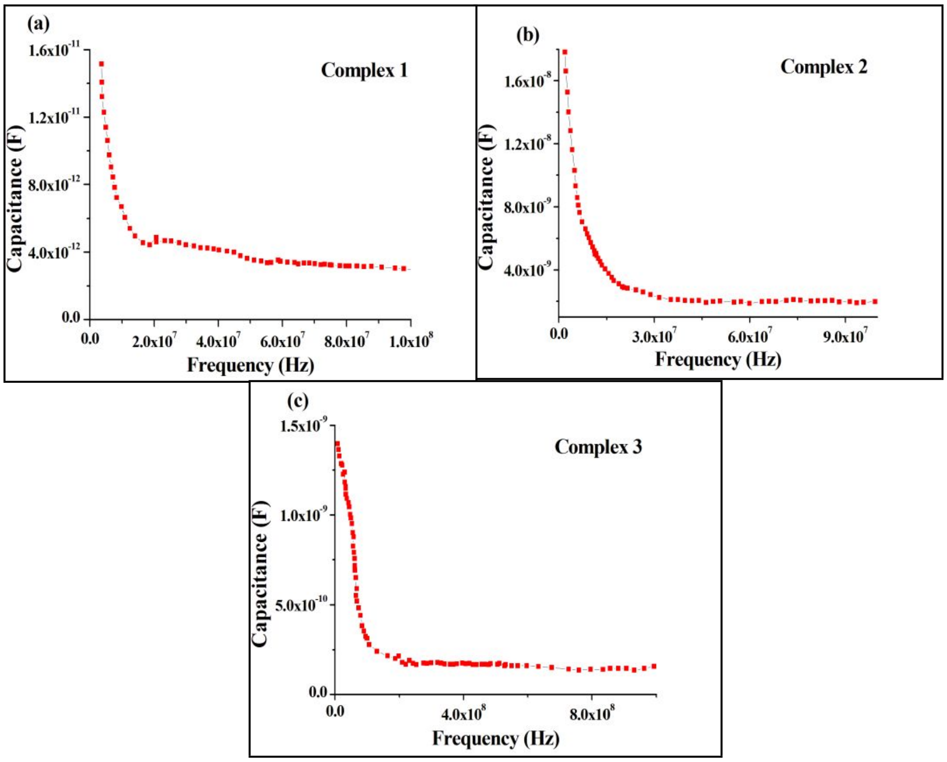

Figure S27. Capacitance *vs.* frequency plots of complexes **1**, **2** and **3** (a, b and c respectively).

Table S6

Device	condition	On /off	conductivity δ (S m^{-1}) $\times 10^{-4}$	photosensitivity	ideality factor η	dv/dlnI Rs (k ohm)	H Rs (k ohm)
1	dark	4.14	7.20		4.1	7.76	13.1
	light	133	15.64	1.78	1.48	5.63	7.67
2	dark	4.54	10.72		4.0	2.76	6.86
	light	165	22.35	2.0	1.39	0.256	4.77
3	dark	4.11	7.89		4.13	6.78	8.22
	light	123	13.5	1.64	1.7	5.33	17.5

Table S7

Device	condition	barrier height Φ_B	mobility μ_{eff} ($cm^2 V^{-1} s^{-1}$)	transit time $t \times 10^{-12}$ (s)	D $\times 10^{-6}$	L_D $\times 10^{-8}$	carrier concentration $N \times 10^{22}$
1	dark	0.89	1.01×10^{-8}	122.71	2.65	2.52	1.63
	light	0.65	1.58×10^{-8}	100.04	4.16	2.89	2.56
2	dark	0.76	10.5×10^{-4}	112.23	2.76	7.87	1.54
	light	0.43	17.8×10^{-4}	31.60	27.1	13.09	2.94
3	dark	0.96	0.32×10^{-8}	135.63	2.74	2.43	0.94
	light	0.74	1.33×10^{-8}	118.63	6.37	6.33	2.19

Theoretical Methods for Non-covalent Interactions

For the DFT calculations of the supramolecular assemblies we have used the PBE0-D3/def2-TZVP level of theory and the Gaussian-16 program.[S14–S17] The binding energies were computed as the difference between the energy of the assembly and the sum of the isolated monomers. The energies have been corrected for the basis set superposition error.[S18] The MEP surfaces were generated using the 0.001 isosurface to emulate the van der Waals envelop. The QTAIM and NCI Plot analyses were performed and represented at the same level using the AIMAll program.[S19–S21] The NCI plot method is convenient to reveal interactions in real space. It uses the reduced density gradient isosurfaces and a colour code (based on the sign of the second eigenvalue of ρ, λ_2) to identify the attractive or repulsive nature of the interactions. The following settings were used in this work: RDG = 0.5, density cut-off = 0.04 a.u., colour scale -0.04 a.u. $\leq \text{sign}\lambda_2(\rho) \leq 0.04$ a.u. Blue and green colours are used here to identify strongly and moderately attractive interactions, respectively.

Theoretical Methods for Optical Properties

The crystals (**1**, **2**, and **3**) were optimized with the DFT method using the CASTEP program code of Accelrys, Inc.[S22] The atomic positions within the crystal were optimized while preserving the experimental unit cell parameters. The optimizations were realized with GGA approximation, PBE functional,[S23,S24] and ultrasoft pseudopotentials[S25] with the relativistic treatment of the Koelling-Harmon.[S26] A plane-wave basis set (340 eV cutoff) was used in the Γ points over the Brillouin zone. The energy tolerance for self-consistent field (SCF) convergence was 1×10-6 eV/atom for geometric optimization. The Tkatchenko-Scheffler scheme of DFT-D dispersion correction has been included in the calculations.[S27] Band Structures and Total and Partial density of states were calculated with HS03 hybrid functional.[S28] Band structures follow the k-vector of the first Brillouin zone of the crystals, and the Total and Partial density of states were plotted concerning the Fermi level with a 2X2X2 grid. Norm-conserving pseudopotentials[S22] were used to join a plane-wave basis set (750 eV cutoff) in the G points over the Brillouin zone. The optical properties, including dielectric function and optical conductivity of the crystal, are calculated for plane-polarized light with the polycrystalline approximation. This calculation was realized with GGA approximation, PBE functional[S23,S24] and norm-conserving pseudopotentials[S28] with the relativistic treatment of the Koelling-Harmon.[S26] The final representations were corrected using the scissors operator until the values were obtained with the HSE03 hybrid functional for calculating bands and PDOS. The smearing of 0.2 eV was employed. Molecular orbital calculations used double numerical with polarization (DNP) basis set[S29–S31] in the all-electron scheme, Γpoint set, and PBE functional[S23,S24] using the Dmol3 program code of Accelrys, Inc.[S22]

References

(S1) *Bruker, SMART v5.631*, Bruker AXS Inc., Madison, WI, USA, 2001.

(S2) Sheldrick, G. M. SHELXT-Integrated Space-Group and Crystal-Structure Determination. *Acta Crystallogr., Sect. A: Found. Adv.***2015**, *71*, 3−8.

(S3) Sheldrick, G. M. SHELXL-2016/6, Program for Crystal Structure Solution; University of Göttingen, 2016.

(S4) Holder, C.F.; Schaak, R.E. Tutorial on Powder X-Ray Diffraction for Characterizing Nanoscale Materials. *ACS Nano* **2019**, *13*, 7359–7365.

(S5) Zhao, H.; Zhang, B. G.; Zhang, X. X.; Wang, S.; Wu, B. L. Homochiral ZnII–Camphorate Frameworks with 4′-p-tolyl-2, 2′: 6′, 2 ″-Terpyridine and 1, 10-Phenanthroline as Accessorial Ligands: Syntheses, Crystal Structures, and Properties. *Synth. React. Inorg., Met.-Org., Nano-Met. Chem.***2015**, *45*, 572–580.

(S6) Spackman, M.A.; Jayatilaka, D. Hirshfeld Surface Analysis. *CrystEngComm* **2009**, *11*, 19−32.

(S7) Hirshfeld, F.L. Bonded-Atom Fragments for Describing Molecular Charge Densities. *Theor. Chim. Acta* **1977**, *44*, 129−138.

(S8) Clausen, H. F.; Chevallier, M. S.; Spackman, M.A.; Iversen, B. B. Three New Co-Crystals of Hydroquinone: Crystal Structures and Hirshfeld Surface Analysis of Intermolecular Interactions.*New J. Chem.* **2010**, *34*, 193–199.

(S9) Rohl, A. L.; Moret, M.; Kaminsky, W.; Claborn, K.; McKinnon, J.J.; Kahr, B.Hirshfeld Surfaces Identify Inadequacies In Computations of Intermolecular Interactions In Crystals: Pentamorphic 1, 8-Dihydroxyanthraquinone.*Cryst. Growth Des.* **2008**, *8*, 4517–4525.

(S10) Parkin, A.; Barr, G.; Dong, W.; Gilmore, C. J.; Jayatilaka, D.; McKinnon, J. J.; Spackman, M.A.; Wilson, C. C. Comparing Entire Crystal Structures: Structural Genetic Fingerprinting. *CrystEngComm* **2007**, *9*, 648–652.

(S11) Spackman, M. A.; McKinnon, J. J. Fingerprinting Intermolecular Interactions in Molecular Crystals. *CrystEngComm* **2002**, *4*, 378–392.

(S12) Spackman, P. R.; Turner, M. J.; McKinnon, J. J.; Wolff, S. K.; Grimwood, D. J.; Jayatilaka, D.; Spackman, M. A. CrystalExplorer: A Program for Hirshfeld Surface Analysis, Visualization and Quantitative Analysis of Molecular Crystals.*J. Appl. Cryst.***2021**, *54*, 1006–1011.

(S13) Clark, S. J.; Segall, M. D.; Pickard, C. J.; Hasnip, P. J.; Probert, M. I.; Refson, K.; Payne, M. C. First Principles Methods Using CASTEP. *Z. Kristallogr.* **2005**, *220*, 567−570.

(S14) Adamo, C.; Barone, V. Toward Reliable Density Functional Methods without Adjustable Parameters: The PBE0Model. *J. Chem. Phys.* **1999**, *110*, 6158.

(S15) Grimme, S.; Antony, J.; Ehrlich, S.; Krieg, H. A Consistent and Accurate Ab Initio Parametrization of Density Functional Dispersion Correction (DFT-D) For The 94 Elements H-Pu. *J. Chem. Phys.* **2010**, *132*, 154104.

(S16) Weigend, F.; Ahlrichs, R. Balanced Basis Sets of Split Valence, Triple Zeta Valence and Quadruple Zeta Valence Quality for H to Rn: Design and Assessment of Accuracy. *Phys. Chem. Chem. Phys.* **2005**, *7*, 3297−3305.

(S17) Frisch, M. J.; Trucks, G. W.; Schlegel, H. B.; Scuseria, G. E.; Robb, M. A.; Cheeseman, J. R.; Scalmani, G.; Barone, V.; Petersson, G. A.; Nakatsuji, H.; Li, X.; Caricato, M.; Marenich, A. V.; Bloino, J.; Janesko, B. G.; Gomperts, R.; Mennucci, B.;

Hratchian, H. P.; Ortiz, J. V.; Izmaylov, A. F.; Sonnenberg, J. L.; Williams-Young, D.; Ding, F.; Lipparini, F.; Egidi, F.; Goings, J.; Peng, B.; Petrone, A.; Henderson, T.; Ranasinghe, D.; Zakrzewski, V. G.; Gao, J.; Rega, N.; Zheng, G.; Liang, W.; Hada, M.; Ehara, M.; Toyota, K.; Fukuda, R.; Hasegawa, J.; Ishida, M.; Nakajima, T.; Honda, Y.; Kitao, O.; Nakai, H.; Vreven, T.; Throssell, K.; Montgomery, J. A. J.; Peralta, J. E.; Ogliaro, F.; Bearpark, M. J.; Heyd, J. J.; Brothers, E. N.; Kudin, K. N.; Staroverov, V. N.; Keith, T. A.; Kobayashi, R.; Normand, J.; Raghavachari, K.; Rendell, A. P.; Burant, J. C.; Iyengar, S. S.; Tomasi, J.; Cossi, M.; Millam, J. M.; Klene, M.; Adamo, C.; Cammi, R.; Ochterski, J. W.; Martin, R. L.; Morokuma, K.; Farkas, O.; Foresman, J. B.; Fox, D. J. Gaussian 16, Revision A.01; Gaussian, Inc.: Wallingford, CT, 2016.

(S18) Boys, S. F.; Bernardi, F. The Calculation of Small Molecular Interactions by the Differences of Separate Total Energies. Some Procedures with Reduced Errors. *Mol. Phys.* **1970**, *19* (4), 553−566.

(S19) Bader, R. F. W. A Quantum Theory of Molecular Structure and Its Applications. *Chem. Rev.* **1991**, *91*, 893−928.

(S20) Contreras-Garcia, J.; Johnson, E. R.; Keinan, S.; Chaudret, R.; Piquemal, J. P.; Beratan, D. N.; Yang, W. NCIPLOT: a program for plotting non-covalent interaction regions. *J. Chem. Theory Comput.* **2011**, *7*, 625−632.

(S21) Lu, T.; Chen, F. Multiwfn: A Multifunctional Wavefunction Analyzer. *J. Comput. Chem.* **2012**, *33*, 580−592.

(S22) Perdew, J. P.; Burke, K.; Ernzerhof, M. Generalized Gradient Approximation Made Simple. *Phys. Rev. Lett.* **1996**, *77*, 3865−3868.

(S23) Perdew, J. P.; Chevary, J. A.; Vosko, S. H.; Jackson, K. A.; Pederson, M. R.; Singh, D. J.; Fiolhais, C. Atoms, Molecules, Solids, And Surfaces: Applications of The Generalized Gradient Approximation for Exchange and Correlation. *Phys. Rev. B.* **1992**, *46*, 6671−6687.

(S24) Vanderbilt, D. Soft Self-Consistent Pseudopotentials in A Generalized Eigenvalue Formalism. *Phys. Rev. B* **1990**, *41*, 7892−7895.

(S25) Koelling, D. D.; Harmon, B. N. A Technique for Relativistic Spin-Polarised Calculations. *J. Phys. C: Solid State Phys.* **1977**, *10*, 3107−3114.

(S26) Tkatchenko, A.; Scheffler, M. Accurate Molecular Van der Waals Interactions from Ground-State Electron Density and Free-Atom Reference Data. *Phys. Rev. Lett.* **2009**, *102*, 073005.

(S27) Heyd, J.; Scuseria, G. E.; Ernzerhof, M. "Hybrid Functionals Based on A Screened Coulomb Potential". *J. Chem. Phys.* **2003**, *118*, 8207−8215.

(S28) Delley, B. An All-Electron Numerical Method for Solving the Local Density Functional for Polyatomic Molecules. *J. Chem. Phys.* **1990**, *92*, 508.

(S29) Delley, B. Fast Calculation of Electrostatics in Crystals and Large Molecules. *J. Phys. Chem.* **1996**, *100*, 6107.

(S30) Delley, B. From molecules to solids with the DMOL3 approach. *J. Chem. Phys.* **2000**, *113*, 7756.

(S31) Slabbert, C.; Rademeyer, M. Structures and Trends of Neutral MX_X $Solvent_{4-X}$ Tetrahedra and Anionic $[MX_4]^{2-}$ Tetrahalometallates of Zinc (II), Cadmium (II) And Mercury (II) with Benzopyridine-and Benzopyrazine-Type N-Donor Ligands or Cations. *CrystEngComm* **2016**, *18*, 4555−4579.
